# Can large language models predict public perceptions of support for climate action across 125 countries?

**DOI:** 10.1093/pnasnexus/pgag374

**Published:** 2026-10-31

**Authors:** Nattavudh Powdthavee, Sandra J Geiger

**Affiliations:** Division of Economics, Nanyang Technological University, 48 Nanyang Avenue, Singapore 639798, Singapore; Behavioral Science for Policy Lab, Princeton University, Princeton, NJ 08540, USA

**Keywords:** pluralistic ignorance, large language models, climate change perceptions, social projection, second-order beliefs, computational social science

## Abstract

Second-order beliefs (“what people think others think”) are theorized to be a powerful driver of climate action, yet they remain costly and difficult to measure at a global scale. Here, we tested whether large language models (LLMs) can predict second-order beliefs about willingness to contribute financially to address climate change across 125 countries. In a preregistered experiment, we prompted four contemporary LLMs and benchmarked their predictions against nationally representative samples from the Gallup World Poll 2021/2022 and statistical regression models. Among the LLMs, Claude and Llama achieved the lowest average errors (∼5 and 7 percentage points, respectively). Claude's error was not statistically different from those of ordinary least squares and Lasso, whereas Llama, GPT, and Gemini performed significantly worse. Yet, accuracy depended strongly on whether prompts included each country's actual first-order willingness to act. Without it, correlations between predicted and observed second-order beliefs were close to zero across all models, even when demographic, economic, and climate information was provided. In exploratory analyses, Claude estimated first-order willingness from country-level information and used these estimates to predict second-order willingness; however, the resulting second-order predictions were unrelated to the observed values. Counterfactual and memorization checks found little evidence that the models were retrieving memorized values; the models appear mainly to map the supplied first-order value onto a second-order estimate, with little contribution from the other country-level information. Overall, the tested approach cannot substitute for survey measurement in settings where first-order data are unavailable.

Significance Statement Addressing climate change requires coordinated action, yet people systematically underestimate how many others are willing to act, a perception gap that suppresses engagement and fuels collective inaction. Measuring these gaps globally is costly, requiring paired surveys from representative national samples. We show that some large language models can predict second-order willingness to act across 125 countries with relatively low average error, but only when provided with each country's actual first-order willingness to act. Without this information, predictions show no relationship with observed values. Replacing the real first-order measure with a large language mode's own estimate also produces inaccurate second-order predictions. Currently, the tested method cannot replace survey measurement when first-order beliefs are unavailable.

Addressing climate change requires rapid, coordinated changes in individual behavior and in broader social and political systems (1, 2). Recent opinion polls indicate strong public support: Worldwide, 69% are willing to contribute 1% of their income to help address climate change, and 89% call for more ambitious government action (3). Yet despite this support, current mitigation efforts remain insufficient (4, 5). Multidisciplinary research on cooperation suggests that progress on climate action depends less on personal preferences than on *beliefs* about others’ support and actions (6–12). However, these second-order beliefs are often inaccurate (pluralistic ignorance (13–15)). People consistently underestimate how many others agree that anthropogenic climate change is real (16, 17), are concerned about it (18–21), support transformative climate policies (22–27), and engage in mitigation behaviors (28–30). For example, even though a global supermajority is willing to contribute a share of their income to climate action, people believe that only 43% are willing to do so (3).

Such underestimations are theorized to be a key barrier to climate action. Because individuals are more likely to contribute to collective action when they believe others are doing so (conditional cooperation) (31–33), misperceptions can suppress expressed support (17, 19, 34) and action, reinforcing a self-perpetuating spiral of silence and inaction (35–37). Correcting misperceptions can help reverse this dynamic and stimulate climate discussion and engagement (3, 36). Although the evidence is mixed (10, 17, 19, 27, 38–41), interventions tend to be most effective where perception gaps are large (42–44). Designing likely effective interventions, therefore, requires timely global maps of perception gaps. Yet large-scale measurement remains costly and logistically challenging, requiring paired measures of personal and perceived attitudes from high-quality, representative samples.

Large language models (LLMs) offer a potential way to infer second-order beliefs at scale, but whether they can do so with sufficient accuracy remains an open question. Recent work on large language model (LLM) forecasting of *first*-order beliefs (eg subjective well-being (45), political opinion (46–51), and attitudes toward climate change (52) and mitigation policies (53)) suggests mixed accuracy, with substantial cross-national disparities, where performance is typically strongest in Western, English-speaking, and higher-income countries (47), consistent with broader evidence that LLMs default to homogenized, Western-skewed representations of non-default cultural groups (54). By contrast, evidence that LLMs can capture second-order beliefs remains limited to controlled settings (55, 56). Predicting second-order beliefs is more demanding than predicting first-order attitudes because it requires estimating how people perceive others, rather than simply what people themselves believe (57). In the human literature, second-order beliefs are thought to arise partly through social projection: people infer others’ attitudes by anchoring on their own without adjusting enough, which typically produces a systematic, directional bias (58). Whether LLMs can do this reliably remains an open question.

This question also has practical implications. If LLMs can reliably infer second-order beliefs from limited information, LLM-based forecasts can help address a genuine and costly measurement gap, particularly where fielding paired surveys is least feasible. But treating LLM outputs as a substitute for direct measurement carries its own risks. Uncritical reliance on artificial intelligence-generated estimates creates an illusion of understanding that outpaces the underlying evidence (59), a risk that is especially consequential in cross-cultural and low-resource contexts, where premature adoption could misallocate scarce survey resources or reinforce existing data inequalities rather than correct for them (60). These estimates should not be used in practice until their accuracy and data requirements are clear.

Here, we test LLM predictions against country-level survey estimates of second-order climate beliefs across 125 countries. In a preregistered experiment, we prompted four leading LLMs—GPT-4o mini, Claude 3.5 Haiku, Gemini 2.5 Flash, and Llama 4 Maverick (17B)—to estimate public perceptions of others’ willingness to contribute financially to address climate change on a 0 (no one) to 100% (everyone) scale. LLMs generated predictions under eight input conditions (stages 1–8), systematically varying country-level sociodemographic, macroeconomic, and climate-related information. We benchmarked their performance against (i) “ground truth” perception gaps observed in 125 countries in the Global Climate Change Survey (GCCS) (3) (*n* = 129,902 respondents; *Md* = 1,000 per country) from the Gallup World Poll 2021/2022 using confirmatory error-based and exploratory correlation-based evaluation and (ii) ordinary least squares (OLS) and Lasso models, using out-of-sample evaluation.

We further conducted non-preregistered experiments to probe how LLMs generate predictions, assess generalizability across prompt formats, and test performance across diverse US climate policies, including a test that isolates which specific inputs drive prediction accuracy by removing them one at a time. We also ran three additional checks to assess whether predictions respond to the supplied inputs or reflect retrieval of survey-specific values: (i) directly asking the models to recall the data, (ii) comparing model versions trained before versus after the source data were published, and (iii) testing whether models with more opportunity to have seen the data perform better. Finally, to test whether this capability generalizes beyond settings with real survey data, we used a Claude-based cascade rather than survey-measured first-order beliefs as the input to second-order prediction. We also extended the design to a non-Western-developed model to test whether training origin shapes prediction accuracy.

In short, our analyses test whether LLMs can estimate second-order beliefs across countries, what information they require, and whether their predictions can be used when survey data are unavailable.

## Results

### Predicting perception gaps across 125 countries using LLMs

We first map perception gaps about climate action using the GCCS (3) (Figure 1A) and compare them with LLM-based estimates (Figure 1B). Consistent with prior work (3), perception gaps are widespread: respondents underestimate others’ willingness to contribute 1% of their household income by an average of 28.70 percentage points (SD = 8.65 p.p.; range 2.71–43.96 p.p.; 95% CI [27.17, 30.24]), largest in West Africa and South Asia (eg Guinea: 44.29 p.p., Afghanistan: 41.50 p.p.) and smallest in Western Europe (eg France: 12.59 p.p.; full values in Figure 1A). In line with social projection, actual and perceived willingness are strongly correlated at the country level (as reported in Andre et al. (3); *ρ* = 0.73; 95% CI [0.64, 0.81]). Actual willingness varies substantially more across countries (SD = 12.51 p.p.) than perceived willingness of others (SD = 7.40 p.p.), a variance ratio of roughly 3:1. Guinea and Afghanistan report far higher personal willingness than France (83.6 and 82.0% vs 51.1%), while perceived willingness of others is comparable across all three (39.7%, 40.5%, 38.1%). These examples illustrate how similar perceptions of others’ willingness can coexist with substantial cross-country differences in actual willingness.

**Figure 1:**
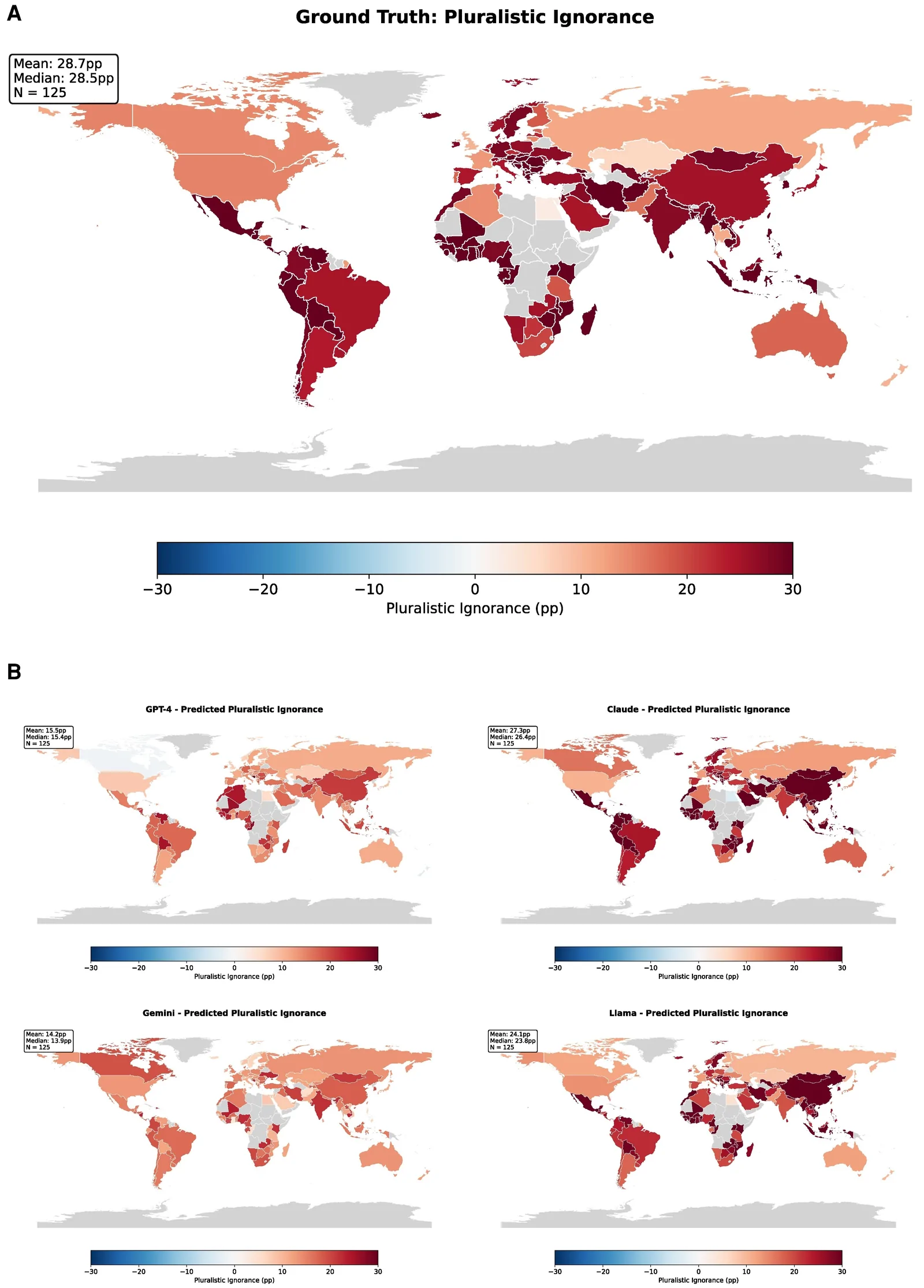
Observed and LLM-predicted global perception gaps. A) Ground-truth unweighted country-level perception gaps (personal willingness to contribute financially to address climate change minus perceptions of others’ willingness), based on 125 countries in the Global Climate Change Survey as part of the Gallup World Poll 2021/2022. B) LLM-estimated perception gaps, computed as observed personal willingness minus each LLM’s predicted second-order belief (perceived others’ willingness), using country names, average sociodemographic, macroeconomic, and climate-related variables, and observed personal willingness (stage 8) as input; LLMs predicted second-order beliefs directly and did not predict the gap itself.

All LLMs recover the broad spatial structure of these perception gaps (Figure 1B) given full contextual information (stage 8: first-order willingness, sociodemographic, macroeconomic, and climate-related data), though predicted levels differ substantially across models. We evaluate performance in terms of both level accuracy (mean absolute error [MAE]: how close each predicted percentage is to the observed value) and cross-country differentiation (Pearson's *r*: whether predicted differences across countries match observed differences). Claude shows the lowest prediction error (MAE = 5.06 p.p. [4.38, 5.82]) and closely matches the observed global mean of second-order belief (41.31 p.p.; SD = 7.40 p.p.; range 19.43–64.67 p.p.), despite only moderate correlation with observed values (Pearson's *r* = 0.51 [0.38, 0.62]). Llama performs similarly on error (MAE = 6.83 p.p. [6.04, 7.68]; *r* = 0.55 [0.41, 0.67]), while GPT and Gemini show substantially larger errors (GPT: MAE = 13.60 p.p. [12.52, 14.67]; *r* = 0.70 [0.60, 0.79]; Gemini: MAE = 14.92 p.p. [13.46, 16.38]; *r* = 0.70 [0.61, 0.77]). The two metrics therefore give different views of model performance; see Figure S1 in the SI. Claude compresses toward the global mean (SD = 3.94 p.p., about half the true SD of 7.40 p.p.; range 28.5–47.6 p.p. vs the true 19.4–64.7 p.p.), lowering absolute error but attenuating correlation; GPT and Gemini show greater spread but systematic bias instead (GPT: SD = 9.79 p.p., range 25.0–75.0 p.p.; Gemini: SD = 12.66 p.p., range 22.5–80.9 p.p.). Llama is not compressed this way (SD = 7.18 p.p., range 25.4–64.4 p.p.); its error instead reflects a consistent upward shift. Consistent with these findings, Spearman's *ρ* as a rank-based metric is lower for Claude and Llama (0.47 [0.31, 0.61], 0.52 [0.36, 0.65]) than for GPT and Gemini (0.70 [0.59, 0.79], 0.71 [0.61, 0.79]; Kendall's *τ* = 0.35 [0.23, 0.46], .40 [0.27, 0.51], 0.56 [0.46, 0.65], 0.52 [0.44, 0.60], respectively), though the confidence intervals for Claude and GPT overlap, indicating this separation is directional rather than sharp.

For benchmarking against traditional statistical and machine learning models, we used repeated 80:20 train-test splits over 10 iterations, stratified by continent, restricted to the *n* = 113 countries with complete data on all covariates, including first-order willingness (a smaller sample than the *N* = 125 used elsewhere, since OLS and Lasso require no missing values). OLS and Lasso significantly outperformed Llama, GPT, and Gemini (all *P* < 0.0001), and outperformed Claude numerically but not significantly (OLS: *t*(9) = 2.02, *P* = 0.074; Lasso: *t*(9) = 1.95, *P* = 0.084). OLS achieved the lowest test error (MAE = 3.90 p.p. [3.56, 4.25]), matched closely by Lasso (MAE = 3.90 p.p. [3.49, 4.31]; see Figure S2, Panel A in the SI). On this same *n* = 113 subsample, Claude's error was 4.48 p.p. (3.66, 5.30) (compared with 5.06 p.p. [4.38, 5.82] on the full *N* = 125 sample), Llama's was 6.86 p.p. (5.86, 7.85), and GPT and Gemini showed substantially larger errors (13.07 p.p. [12.11, 14.03] and 14.01 p.p. [12.77, 15.24], respectively). These conclusions also hold for root mean squared error (RMSE) (Figure S2, Panel B).

To test whether Claude's low predicted error from using all available country-level information reflects genuine country-level differentiation, or whether it is comparable to what a trivial guess would achieve, we compared it to the simplest possible reference point: predicting the same number, 41.31 (the average value across all 125 countries), for every country regardless of which one it is. Averaged across all 125 countries, this single fixed guess is off by 5.44 p.p. (4.55, 6.36), barely worse than Claude's MAE of 5.06 p.p. (4.37, 5.81). A regression using only first-order willingness does even better (4.03 p.p. [3.56, 4.49]) than Claude. Claude's accuracy, therefore, does not clearly exceed that of a guess that makes no distinction between countries at all, and falls short of that of a model using a single country-specific variable. These comparisons thus raise the question of whether LLM predictions reflect a broader projection process or simply extrapolate from the supplied first-order data.

### How do LLMs infer public perceptions of others’ climate action?

Having shown that Claude achieved the lowest prediction error for second-order belief among the four LLMs tested, but that its predictions were not substantially better than a constant global-mean prediction, we examine how these predictions are generated. We first ask which country-level features drive LLM predictions and whether these match the empirical predictors of human second-order beliefs. We then test which supplied inputs actually matter by varying them systematically and removing them one at a time.

### Which predictors of public perceptions of others’ climate action do LLMs represent accurately?

Using OLS regressions, we find that people in countries with higher mean willingness to contribute financially to climate action (*b* = 0.79, SE = 0.08, *P* < 0.001) and higher inequality (*b* = 0.13, SE = 0.07, *P* = 0.043) report stronger perceptions of others’ willingness to contribute. All other country-level predictors are nonsignificant (Table 1). Regressing each LLM's predictions on the same features shows that Claude and Llama's outputs closely match the true coefficient on the first-order willingness (*b* = 0.79 and 0.84), whereas GPT and Gemini substantially overweight it (*b* = 0.96 and 0.95) (58). These regressions describe the correlational structure of each model’s output; they do not establish functional reliance, since features correlated with first-order willingness can appear as directionally correct predictors even if a model never uses them directly. We test this distinction in the analyses below by systematically varying which information LLMs are given.

**Table 1:** OLS regressions predicting perceptions of others’ climate action: ground truth versus LLMs (stage 8 with all predictors).

	(1)	(2)	(3)	(4)	(5)
	Actual	GPT	Claude	Gemini	Llama
Mean personal willingness to contribute financially to address climate change	0.788^b^	0.961^b^	0.787^b^	0.954^b^	0.836^b^
	(0.081)	(0.061)	(0.094)	(0.033)	(0.057)
Mean age	−0.095	−0.088	−0.108	−0.039	−0.031
	(0.114)	(0.059)	(0.078)	(0.038)	(0.065)
Mean education level	0.065	0.154*	0.022	0.100*	0.018
	(0.090)	(0.066)	(0.094)	(0.045)	(0.092)
Mean religiosity	−0.111	0.044	0.104	0.033	0.077
	(0.087)	(0.061)	(0.085)	(0.040)	(0.086)
HDI index (2021)	0.123	0.031	0.178	−0.040	0.007
	(0.084)	(0.061)	(0.112)	(0.060)	(0.125)
GDP per capita (2021)	−0.089	0.106	0.138	0.049	0.204*
	(0.080)	(0.072)	(0.087)	(0.042)	(0.097)
Share of income held by the top 1% (2021)	0.133^a^	−0.013	0.030	−0.047	−0.065
	(0.065)	(0.044)	(0.072)	(0.033)	(0.067)
Average temperature 2010–2019	−0.078	−0.004	0.071	0.002	0.024
	(0.097)	(0.059)	(0.103)	(0.040)	(0.073)
Constant	0.000	−0.000	−0.000	−0.000	−0.000
	(0.059)	(0.038)	(0.059)	(0.031)	(0.056)
Observations	125	125	125	125	125
*R* ^2^	0.591	0.827	0.587	0.884	0.635
Adjusted *R*^2^	0.563	0.815	0.559	0.876	0.610

SEs in parentheses.

^a^ *P* < 0.05.

^b^ *P* < 0.01.

### Which information do LLMs require to predict public perceptions of others’ climate action?

To identify the information LLMs use to predict public perceptions of others’ climate action, we systematically vary the inputs provided to the LLMs and examine how prediction accuracy responds, evaluating it using both MAE and correlation with the ground truth. We first add information in stages (*sequential information addition*), then remove individual inputs from the full prompt (*feature ablation*), and finally alter key inputs while holding the others fixed (*counterfactual tests*). Together, these analyses show which information drives the models’ predictions and whether country names alone prompt them to reproduce stored survey values.

#### Sequential information addition (confirmatory: MAE; exploratory: correlations)

Results above used the full specification (stage 8), which includes country-level sociodemographic (age, education, religiosity), macroeconomic (gross domestic product [GDP], inequality, human development index [HDI]), and climate-related (temperature, personal willingness) information. To identify which inputs improve LLM predictions, we add information sequentially (Figure 2A). With only country names (stage 1), MAE is relatively low for three of four models (Claude: 7.56 p.p. [6.38, 8.83]; Llama: 8.12 p.p. [7.07, 9.28]; GPT: 8.99 p.p. [7.88, 10.13]; Gemini: 13.0 p.p. [11.49, 14.60]). This low error, however, does not reflect genuine country-level accuracy: correlation with ground truth at stage 1 is close to zero for every model (Claude: *r* = −.11 [−0.35, 0.13]; Llama: *r* = +0.10 [−0.09, 0.26]; GPT: *r* = −0.12 [−0.29, 0.06]; Gemini: *r* = +0.04 [−0.13, 0.21]; Figure 2B), and remains near zero through stages 2–4 and 6–7, regardless of which sociodemographic, macroeconomic, or climate variables are added.

**Figure 2:**
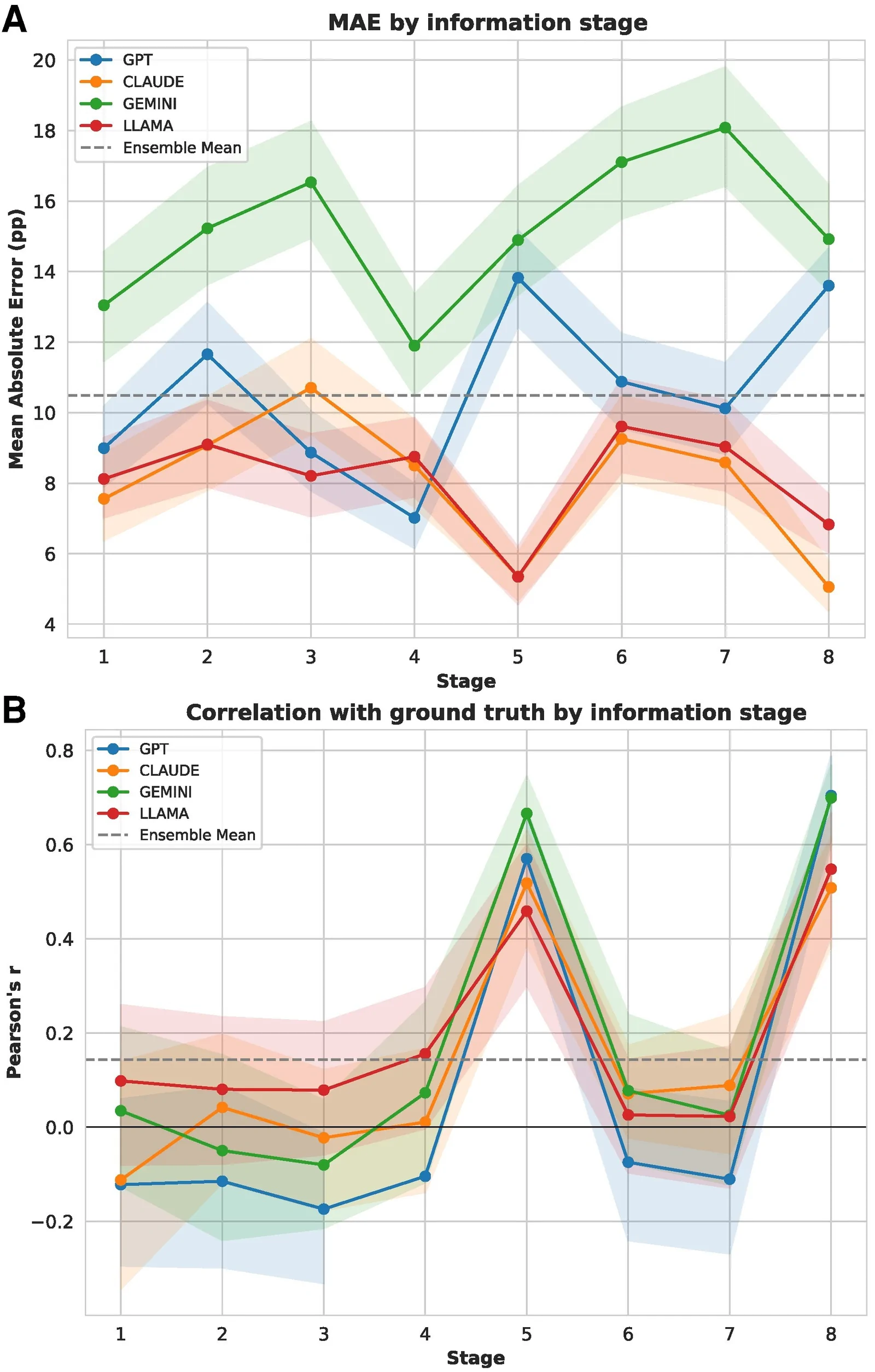
MAE and Pearson's *r* by information stage. A) Mean absolute error (MAE, percentage points); B) Pearson's *r* between predicted and actual second-order beliefs, both for each LLM's predictions of others’ willingness to act on climate change across eight information stages. Stage 1 includes only the country name; stage 2 adds sociodemographic information; stage 3 adds macroeconomic indicators; stage 4 adds temperature data; stage 5 adds first-order willingness to act; stage 6 combines sociodemographic and macroeconomic information; stage 7 adds temperature to stage 6; stage 8 includes all available information. The horizontal dashed line in each panel shows the ensemble mean (across all four LLMs and all eight stages) for that panel's metric. Lower values indicate more accurate predictions in (A); values further from zero indicate stronger correlation with ground truth in (B). *N* = 125 countries × 8 stages = 1,000 predictions per LLM. Shaded areas show bootstrap 95% confidence intervals (3,000 resamples).

Both MAE and correlation change substantially only at stages 5 and 8, both of which include first-order willingness: MAE drops to its lowest values for Claude and Llama at stage 5 (5.35 p.p. each, [4.67, 6.07] and [4.55, 6.17]) while correlation rises substantially (Claude: *r* = 0.52 [0.39, 0.63]; Llama: *r* = 0.46 [0.29, 0.60]), and GPT and Gemini’s overweighting of this input (Table 1) drives their error up (GPT: 13.82 p.p. [12.50, 15.09]; Gemini: 14.89 p.p. [13.39, 16.42]) even as their correlation also rises (GPT: *r* = 0.57 [0.45, 0.68]; Gemini: *r* = 0.67 [0.57, 0.75]). Across every other stage, adding country-level context that excludes first-order willingness leaves both error and correlation essentially unchanged. We examine what this dissociation implies about how LLMs generate these predictions in the feature ablation and counterfactual tests below.

#### Feature ablation (exploratory)

While adding first-order information improves predictions, the sequential design cannot determine whether this improvement depends on existing features. Feature ablation addresses this by removing each variable individually from the full specification. In MAE terms (Figure 3A), Claude and Llama show little sensitivity to most removals: errors stay within a narrow range (Claude: 5.07 p.p. [4.37, 5.79] to 5.28 p.p. [4.57, 6.06]; Llama: 5.41 p.p. [4.76, 6.11] to 5.89 p.p. [5.20, 6.58]) whether sociodemographic, macroeconomic, climate, or religion variables are removed, and removing first-order willingness specifically increases error only modestly relative to the full specification. Read on MAE alone, this pattern could suggest the models integrate multiple predictors rather than relying on any single one.

**Figure 3:**
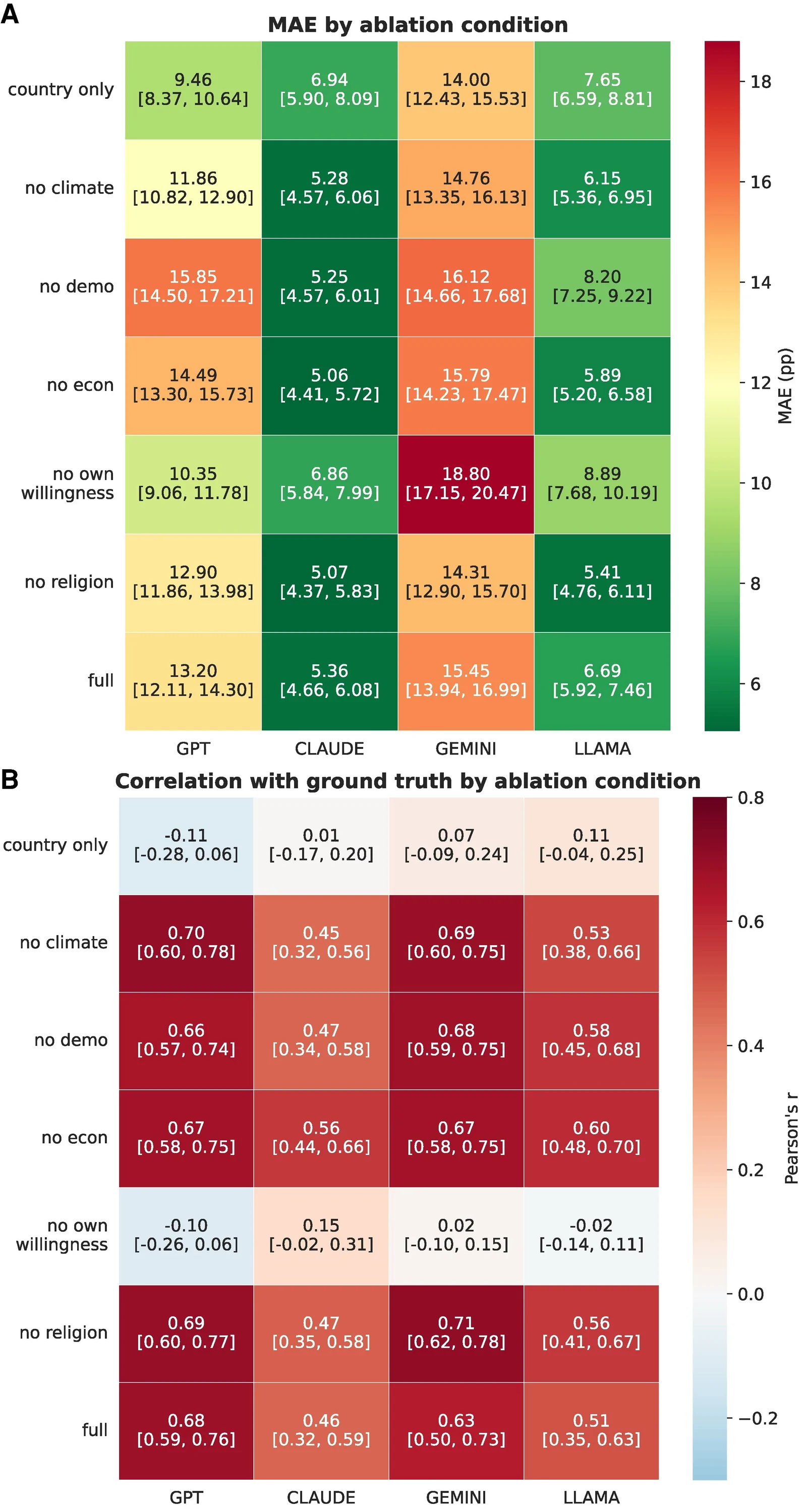
Ablation tests: mean absolute error and correlation under feature removal. A) Heatmap showing mean absolute error (MAE) with 95% CIs for each LLM under single-feature removal from the full specification, and for country name alone (country_only). B) Heatmap showing Pearson’s *r* between predicted and actual second-order beliefs, with 95% CI, for the same ablation conditions. Rows are ordered from least to most information (country_only through full). In (A), larger increases in MAE indicate greater dependence on the removed input. In (B), values near zero indicate no measurable relationship between predictions and ground truth; only removing first-order willingness, or providing country name alone, produces this pattern, while removing any other single feature leaves correlation close to the full-specification value for every model.

Correlation with ground truth (Figure 3B) shows the opposite pattern. Removing any of climate, sociodemographic, macroeconomic, or religion information leaves correlation close to the full-specification value for every model (eg Claude: *r* = 0.45−0.56, vs *r* = 0.47 at full specification). Removing first-order willingness collapses correlation to values statistically indistinguishable from zero for all four models (Claude: *r* = 0.15 [−0.02, 0.32]; Llama: *r* = −0.02 [−0.14, 0.11]; GPT: *r* = −0.10 [−0.27, 0.07]; Gemini: *r* = 0.02 [−0.10, 0.15]), matching the near-zero correlation observed with country name alone. The low MAE after feature removal largely reflects predictions remaining close to the global mean. This provides little evidence that the remaining country-level variables add detectable country-level predictive signal. Only first-order willingness contributes measurably to predictive validity.

GPT and Gemini show a related but distinct failure mode: consistently high MAE across all ablations (Gemini: 14.31–18.80 p.p.; GPT: 9.46–15.85 p.p.), driven by substantially overweighting first-order willingness when it is present (Table 1; GPT: *b* = 0.96, Gemini: *b* = 0.95, vs actual *b* = 0.79). Unlike Claude and Llama, this overweighting inflates error even as correlation remains comparatively high when first-order willingness is available: GPT and Gemini's raw predictions preserve more of the true country-level ordering than Claude's or Llama's, despite their larger average errors, because MAE and correlation penalize different kinds of mistakes.

#### Counterfactual tests (exploratory)

To test whether LLMs infer public perceptions of others’ climate action and their predictors by responding to the information supplied in the prompt or by recalling country-specific values from training, we conducted three deliberate changes to the supplied information (Figure S3 in the SI): (i) reversing personal willingness (high ↔ low), (ii) swapping country names while keeping all features unchanged, and (iii) reversing GDP values (high ↔ low). If LLMs rely on memorization, predictions should follow the country names; if predictions respond to the supplied information, then they should vary when those inputs are changed.

Flipping personal willingness significantly increased MAE for all four models: Gemini (baseline 15.45 p.p. → 19.27 p.p.; +3.81 p.p. [1.70, 5.95], *P* = 0.001), GPT (13.20 → 18.50 p.p.; +5.30 p.p. [3.64, 6.96], *P* < 0.001), Llama (6.69 → 8.27 p.p.; +1.58 p.p. [0.47, 2.77], *P* = 0.009), and Claude, though the increase was smaller for Claude than for the other three models (5.36 → 6.17 p.p.; +0.80 p.p. [0.35, 1.30], *P* = 0.001). Correlation shows the same pattern: flipping willingness reduces Pearson's *r* for every model, including Claude (*r* = 0.47 [0.32, 0.59] to 0.24 [0.08, 0.40]) and Llama (*r* = 0.51 [0.35, 0.63] to 0.06 [−0.13, 0.24]), and drives GPT and Gemini toward zero (GPT: *r* = −0.03 [−0.09, 0.02]; Gemini: *r* = 0.05 [−0.09, 0.20]). Claude retains more correlation than the other models after the flip, but the drop is still substantial, roughly halving *r*, thus providing little evidence that the model is drawing substantially on several inputs at once. Swapping country names and GDP values produces negligible effects on both MAE (all |ΔMAE| < 1.00 p.p.; all *P* > 0.05) and correlation (Figure S3). Predictions are therefore not driven by recall of country-specific values from the country name alone or substantially altered by GDP, but are substantially altered by personal willingness for every model, Claude included. These tests address only one pathway to memorization and do not rule out reliance on survey-specific quantities absorbed during pretraining; we address this directly in the three additional checks for memorization described next.

### Do the models use the supplied information, or recall survey values from training? (exploratory)

We conducted three checks: we asked the models directly to report the published survey values (*direct recall probe*); tested whether models with more opportunity to have encountered the published data performed better (*training cutoff natural experiment*); and compared model versions from before and after the survey was published (*prepublication vs postpublication LLM comparison*).

#### Direct recall probe

We tested whether LLMs can retrieve country-specific values from the GCCS when explicitly prompted, using prompts naming the source (3) and encouraging retrieval, in contrast to the main-study prompt, which instructed models not to rely on memorized survey results. If LLMs had memorized country-specific values, recall accuracy should match or exceed that of stage 8. Instead, recall MAE exceeded stage 8 MAE for Claude (8.02 p.p. [6.75, 8.93] vs 5.06 p.p. [4.36, 5.84]), with no improvement over stage 1 predictions produced under instructions not to use memorized survey results (Figure S4 in the SI). For Claude Haiku 4.5 and Gemini 2.5 Flash, whose training cutoffs postdate GCCS publication, this comparison is not confounded by training cutoff, and both failed to retrieve the data, arguing against memorization for these models. GPT-4o mini and the Llama substitute (Llama 3.3 70B, used in place of Llama 4 Maverick due to endpoint availability) predate publication, so their results are uninformative on this point.

#### Training cutoff natural experiment

Under memorization, later training cutoff should predict lower prediction error, as more postpublication exposure allows more accurate retrieval. We plotted each LLM's training cutoff date against its stage 8 MAE, relative to GCCS publication (paper: February 2024; website: April 2024). The observed rank order is inconsistent with this prediction: GPT-4o mini, the only LLM with a training cutoff predating GCCS publication, does not achieve the lowest MAE, and Gemini 2.5 Flash, with the latest cutoff and maximum post-publication exposure, achieves the highest (Figure S5 in the SI). Because this comparison contains only four models, it is descriptive rather than a formal statistical test of memorization, and, therefore, carries less weight than the recall probe and the prepublication versus postpublication comparison below.

#### Prepublication versus postpublication LLM comparison

We ran stage 8 prompts through prepublication versions of both Claude and Llama, using the same protocol as the main study (Figure S6 in the SI). Prepublication Claude 3 Haiku (cutoff 2023 August, 6 months before GCCS publication) achieved MAE = 7.27 p.p. [6.45, 8.12], *r* = 0.73 [0.63, 0.80], comparable to postpublication Claude Haiku 4.5 (MAE = 8.33 p.p. [7.32, 9.37], *r* = 0.61 [0.49, 0.71]) and to main-study Claude 3.5 Haiku (MAE = 5.06 p.p. [4.38, 5.82], *r* = 0.51 [0.38, 0.62]). The prepublication model marginally outperformed its postpublication counterpart, the opposite of what memorization would predict. The same pattern held for Llama: prepublication Llama 3.3 70B (MAE = 5.18 p.p. [4.50, 5.91], *r* = 0.66 [0.54, 0.75]) performed comparably to, and marginally better than, postpublication Llama 4 Maverick (MAE = 6.83 p.p. [6.04, 7.68], *r* = 0.55 [0.41, 0.66]). Neither family shows the postpublication advantage that memorization would predict; where generational differences exist, they more plausibly reflect changes in general capability than exposure to GCCS data, though the two cannot be fully disentangled without access to training data. Note also that within-model-family variation across versions (Claude: 5.06–8.33 p.p.; Llama: 5.18–6.83 p.p.) is substantially smaller than the between-model variation observed in the main study (5.06–14.92 p.p. across all four LLMs), indicating that model choice matters more than model version for this task.

### Do findings hold across prompt formats and climate policies? (exploratory)

We repeated the full specification using structured prompts (bullet points rather than sentences) with identical information. Results replicate across formats (Figure S7 in the SI): structured prompts yield systematically higher MAE, and removing personal willingness again collapses correlation with ground truth (full *r* = 0.58–0.73 across models; without willingness, *r* = −0.02 to 0.26), while swapping country names and GDP values induces minimal change in either metric.

Restricting the sample to the 112 countries with complete data on all variables, as preregistered, leaves results qualitatively unchanged (Figure S8 in the SI), indicating that handling missing values does not bias predictions.

Finally, we test generalizability across 24 perception gaps regarding public support for a wide range of climate policies (eg carbon tax, renewable energy generation, Green New Deal) using three nationally representative US datasets (21, 23, 61) (Figure S9). Claude (MAE = 8.30 p.p. [6.25, 10.53]) and Llama (MAE = 3.71 p.p. [2.73, 4.78]) again perform best; GPT performs worse (MAE = 17.02 p.p. [14.25, 19.88]); and Gemini produced no predictions for any of the 24 items, apparently due to content filtering of policy-related queries, a limitation for any large-scale application of this approach, discussed further below.

### Can LLMs substitute for missing first-order survey data? (exploratory)

Accurate second-order prediction depends almost entirely on having each country's true first-order willingness. This raises a practical question: could an LLM generate its own estimate of first-order willingness from public country-level data, in place of a real survey, and use that estimate to predict second-order beliefs? Such a two-step cascade would extend this approach to genuinely under-surveyed settings, the use case that motivated interest in it.

Because Claude 3.5 Haiku (claude-3-5-haiku-20241022), used throughout the main study, was retired by Anthropic on 2026 February 19, we conducted this test using Claude Haiku 4.5 (claude-haiku-4-5-20251001). To isolate the accuracy loss from using the model's own first-order estimate from differences between model generations, we benchmarked against the same model given the real first-order value, rather than the main study's stage 5 results.

Claude Haiku 4.5 first estimated each country's first-order willingness using only stage 7 information (no survey data on first-order willingness). These zero-shot estimates showed no relationship to the truth (MAE = 34.73 p.p. [32.03, 37.30], *r* = −0.05 [−0.20, 0.09], *ρ* = −0.10 [−0.27, 0.06]; Figure 4A). Using these estimates, rather than real values, as input to generate second-order prediction collapsed accuracy relative to the same model given the real value (real input: MAE = 8.47 p.p. [7.50, 9.43], *r* = 0.72 [0.64, 0.79]; cascaded: MAE = 17.35 p.p. [15.68, 19.04], *r* = −0.08 [−0.23, 0.08]; paired difference: ΔMAE = +8.88 p.p. [6.93, 10.89], Δ*r* = −0.80 [−0.96, −0.62]; Figure 4B).

**Figure 4:**
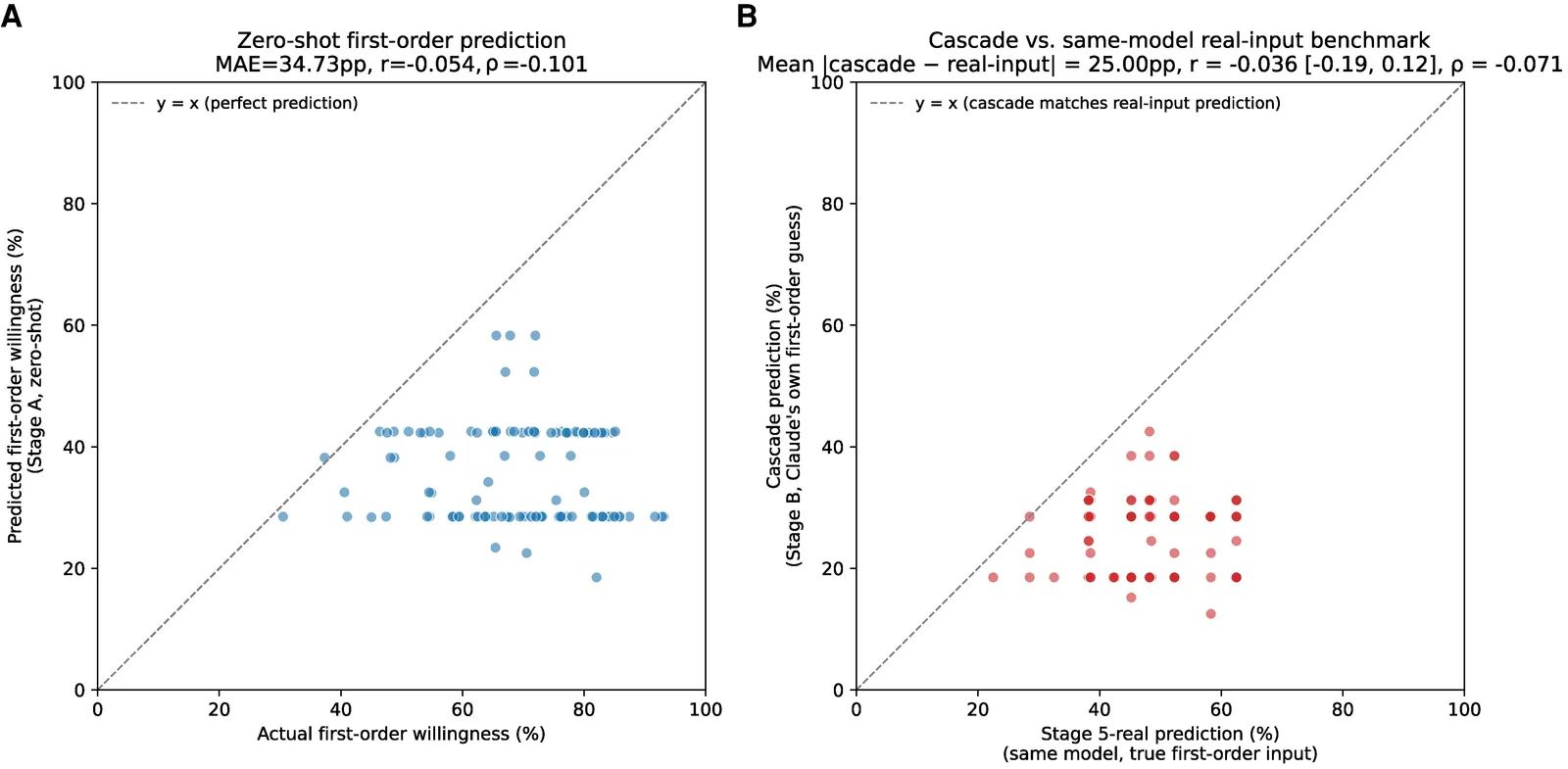
Two-step prediction test and its consequences for the cascade. A) Predicted first-order willingness (stage A, zero-shot, using only stage 7 country-level information) plotted against actual first-order willingness, for Claude Haiku 4.5. The dashed line indicates perfect prediction (*y* = *x*); points clustered away from this line, with no upward trend, indicate no relationship between predicted and actual values (MAE = 34.73 p.p. [32.03, 37.30], *r* = −.05 [−.20, .09]). B) Second-order predictions using Claude's own stage A estimate as input (cascade) plotted against second-order predictions from the same model given the real first-order value (stage 5-real), for the same countries. The dashed line indicates where the two would be identical; the near-total absence of any relationship between the two series (*r* = −0.04, 95% CI [−0.19, 0.12]) shows that cascading substitutes a different, uncorrelated set of predictions rather than a noisier version of the same ones.

The cascade does not extend LLM-based estimation to settings without existing survey data: country-level covariates alone provide no usable signal about first-order willingness, so a model's own estimate does not add noise to a working method; it removes the only input on which second-order prediction depends.

## Discussion

Across 125 countries, Claude and Llama produced the lowest average error (Claude: MAE ≈ 5 p.p.; Llama: ≈ 7 p.p.). However, their performance depended almost entirely on being given each country's real first-order willingness: correlation with ground truth was near zero without it, for every model, and OLS and Lasso regression matched or exceeded LLM accuracy with the same input. An exploratory follow-up test using LLM-generated rather than survey-measured first-order estimates found no relationship between predicted and actual values, and this failure propagated directly into second-order predictions, thereby collapsing accuracy relative to the actual data. The tested approach therefore cannot substitute for survey measurement in under-surveyed settings that would most benefit from a low-cost alternative; a Claude-based cascade analysis showed that even the best-performing model can only refine second-order estimates once first-order data already exist.

We find little evidence that the models retrieved country-specific survey values. Naming the source did not improve recall, and models trained after publication did not outperform earlier versions. One explanation for this is that the GCCS website presents country-level data only via interactive hover and query interfaces rather than downloadable tables (Figure S10 in the SI), thereby limiting its availability for web-based training. Instead, LLMs appear to treat first-order willingness as the dominant, largely exclusive input, extrapolating from it to approximate the true empirical relationship between first-order and second-order beliefs (Table 1). This pattern is more consistent with extrapolating from first-order willingness than with integrating broader country-level information. Predictions from country names alone achieve a superficially low MAE (∼7–8 p.p. for Claude and Llama) but show no reliable correlation with the ground truth, indicating compression toward the sample mean rather than genuine use of country-level information. Whether this extrapolation resembles social projection, as theorized for human second-order beliefs (58), cannot be established from output patterns alone.

### Why do some LLMs succeed where others fail?

GPT and Gemini's common failure mode, systematically overweighting first-order willingness (Table 1), suggests a calibration difference rather than a difference in reasoning depth: Claude and Llama's coefficients on first-order willingness closely match its true empirical relationship with second-order beliefs, while GPT and Gemini treat it as almost the only input that matters for their predictions. The models appear to differ mainly in how they translate first-order into second-order willingness. We find little evidence that the other country-level variables improve their predictions. This matters when choosing a model for settings where first-order data already exist. Nevertheless, it still does not resolve the more basic limitation demonstrated by the cascade analysis (Figure 4): even Claude, the model family that achieves the lowest main-study MAE, could not generate a usable estimate of first-order willingness from the remaining country-level information. A supplementary check extending this design to DeepSeek-V3.1, a Chinese-developed model, shows a partially overlapping pattern: like GPT and Gemini, its error increases sharply once first-order willingness is introduced, but it tracks country rankings more closely than Claude or Llama at full information (Figure S11 in the SI). However, although this single exploratory comparison does not reveal a distinct pattern, it cannot establish whether the model-training origin systematically affects performance.

### Limitations and caution

Our study is not without limitations. First, our analysis operates at the national level and cannot speak to whether these relationships hold at finer scales; national estimates may mask intervention-relevant variation across regions, cities, and demographic groups. Second, all prompts were administered in English. Given that accuracy depends almost entirely on a single supplied value rather than rich country-level representations, the main risk is not that models hold impoverished knowledge of non-English-speaking countries, but that identical numeric information may be processed differently depending on the language of the surrounding prompt, a pattern consistent with broader evidence that LLMs default to homogenized, Western-skewed representations of non-default cultural groups (54, 62). We tested this partially: a single non-Western-developed model (DeepSeek) showed a pattern comparable to, but not identical to, that of the four incumbent models, though this does not constitute a systematic test of training-origin effects (Figure S11). These homogenization risks may be especially important for lower-GDP, non-Western countries, which may be less richly represented in LLM training data. Where prediction errors are larger in such settings, they could reflect both systematic misrepresentation and limited data availability. Previous work has not clearly distinguished between these explanations (47), and the present design cannot do so either. Third, our analysis assumes temporal alignment between LLM training data and Gallup survey fieldwork; a gap of 1–2 years between survey waves and a model's training cutoff could introduce errors that are difficult to disentangle from genuine predictive inaccuracy. Fourth, Gemini 2.5 Flash failed to produce predictions for any of the 24 US climate policy items, apparently due to content filtering of policy-related queries. If leading LLMs apply inconsistent safety filters to policy-relevant questions, this becomes a non-trivial limitation to any large-scale application of this approach across political or cultural contexts.

### Future directions

Future work should first test whether the result generalizes across languages, model families, and subnational populations. Because zero-shot estimation of first-order beliefs failed to produce meaningful predictions, the more promising route to extending this approach to under-surveyed settings may be to use lightweight digital signals, such as search trends, as an alternative source of the anchoring information these models require, rather than asking LLMs to supply that anchor themselves. Where some local survey data already exist, fine-tuning or integration with existing survey infrastructures (eg the Gallup World Poll or regional barometers) may improve calibration and enable subnational estimation. More granular validation across regions, demographic groups, model families, and prompting languages is needed to determine when LLM-generated estimates are sufficiently reliable for resource allocation, and whether the pattern identified here holds for models trained primarily outside the United States (addressed here in an exploratory check, Figure S11) or, more systematically, prompted in languages other than English.

A separate question is whether these estimates improve intervention targeting when first-order survey data are already available. Future work could test whether LLM-generated second-order estimates, in settings where first-order data are available, improve intervention targeting or message framing relative to expert judgment, human forecasters, or simple heuristics—particularly given evidence that intervention effectiveness varies substantially across sociodemographic and national context (63). This question is distinct from whether LLMs can substitute for missing first-order measurement, which the present results argue against.

More generally, second-order beliefs shape collective outcomes well beyond climate action, including public health, cooperation, and political polarization. Whether LLMs depend similarly on observed first-order beliefs in other domains remains unknown.

## Methods

This study examines whether LLMs can predict public perceptions of others’ climate action, and whether observed performance depends on the specific information provided rather than direct recall of survey-specific quantities. We construct country-level measures from the GCCS (3), elicit predictions from multiple LLMs under controlled prompting conditions, benchmark performance against statistical models, and apply a set of diagnostic tests to identify which inputs drive predictions and whether performance could reflect memorization.

The methods follow the computational pipeline implemented in the analysis notebooks: data construction, prompting and inference, evaluation, diagnostic tests, and robustness analyses. The main study was conducted between 2025 October 28 and 2025 November 15; the ablation study was conducted on 2026 January 16; and additional data collection for this revision, including the two-step prediction test, was conducted on 2026 July 12.

### Data sources and outcome construction

Ground-truth data come from the GCCS (3) administered as part of the Gallup World Poll 2021 to 2022, covering 125 countries. The survey measured respondents’ willingness to contribute at least 1% of their household income per month to address climate change, as well as their perceptions of how willing others in their country would be to do so.

All analyses are conducted at the country level, with individual responses aggregated using Gallup sampling weights. Pluralistic ignorance (or perception gaps) is defined as the difference between country-level average first-order willingness and average perceived willingness of others.

The Gallup World Poll conducts independent, nationally representative probability surveys of the civilian, noninstitutionalized population aged 15 and older in each country annually; a fresh sample is drawn each wave rather than recontacting prior respondents, so the design is repeated cross-sectional rather than panel, and panel conditioning does not apply. Data collection uses random-digit-dial telephone interviews in countries where telephone coverage exceeds approximately 80% of the population, and multi-stage stratified area-probability face-to-face interviews elsewhere; a small number of areas are excluded from full national coverage where interviewer safety or extreme remoteness precludes access. Typical sample sizes are approximately 1,000 respondents per country per wave (consistent with *n* = 129,902 across 125 countries here). Gallup applies post-stratification weights, calibrated to national demographic distributions (age, gender, and education, where available), which we use throughout when aggregating individual responses to country-level means. Full country-by-country sampling frames, field periods, and design documentation are available in Gallup's published methodology (https://www.gallup.com/178667/gallup-world-poll-work.aspx).

Auxiliary country-level covariates were unavailable for a small number of countries; missing values were explicitly indicated as unavailable in LLM prompts rather than used to exclude countries. LLM predictions are evaluated on the full sample of 125 countries; analyses that require complete covariate data (eg OLS and Lasso benchmarks, internet-penetration regressions) use the corresponding complete-case subsample, as noted where relevant.

### Country-level inputs

Model inputs consist of country-level demographic, economic, and climate-related factors. Sociodemographic variables (mean age, education, and religiosity) are aggregated directly from the Gallup World Poll 2021/2022 using the same sampling weights as the outcomes.

Macroeconomic indicators (GDP (64), inequality (65), HDI (66)) are drawn from the World Bank and the World Inequality Database; long-run average temperature (2010–2019, °C) from the World Bank Group's Climate Change Knowledge Portal (https://climateknowledgeportal.worldbank.org/download-data) and the CRU TS v.4.05 data (https://crudata.uea.ac.uk/cru/data/hrg/), as reported by Andre and colleagues (3); and internet penetration (% of population) is obtained from World Bank Open Data (World Bank, 2024). All variables are harmonized to the closest available year and merged by country identifier.

None of the inputs contain information on pluralistic ignorance or second-order beliefs.

### Prompting and inference protocol

#### System prompt

All LLMs were queried using a fixed system-level instruction that constrained responses to general reasoning based on the information provided in the prompt and explicitly discouraged reliance on memorized statistics, survey results, or academic sources. This system prompt was identical across LLMs, countries, and the eight main-study prompt stages; the cascade test (see below) used a version with the task description retargeted from second-order to first-order belief prediction, otherwise unchanged. Full verbatim system prompts for both versions are provided in the Supplementary Material (Note S1).

#### User prompt and staged design

Predictions were elicited using a structured user prompt within a staged information framework, ranging from minimal inputs (country name only; stage 1) to a full-specification condition (stage 8). At stage 8, the prompt instructed LLMs to estimate second-order beliefs, defined as respondents’ beliefs about others’ willingness to contribute financially to address climate change, conditional on a specified information set: country name, aggregated sociodemographic characteristics, macroeconomic indicators, long-run average temperature, and the country-level distribution of first-order willingness.

An example of the full-specification prompt for Argentina:

In Argentina, the average age of respondents is 42.1 years, 10.8% of the people have completed a tertiary education, and 52.3% say religion is important in daily life. GDP per capita (PPP, 2021) is $29,484, the top 1% holds 13.0% of total income, and 24.9% of total wealth. The Human Development Index (2021) is 0.842. The average temperature from 2010 to 2019 is 15.3°C. In this survey, 62.2% of people said they are personally willing to contribute 1% of their income each month, and an additional 14.3% would contribute a smaller amount.

In a nationally representative survey with a probability-based sample of approximately 1,000 residents aged 15 and above in Argentina, respondents were asked: “Would you be willing to contribute 1% of your household income every month to fight global warming? This would mean that you would contribute 1 for every 100 of this income.” Responses: Yes, No, (Don’t Know), (Refused). Don’t know and refused were coded as missing data. Respondents were then asked how many respondents in Argentina they think are willing to contribute at least 1% of their household income every month to fight global warming. Responses: between 0% and 100%, (Don’t know), (Refused).

Based on the country, socio-demographic, macro-economic indicators, temperature data, and the actual willingness data shown above, estimate what respondents in Argentina on average thought about how many **other** respondents in Argentina are willing to contribute at least 1% of their household income every month to fight global warming. Note: You are estimating people's **beliefs** about others’ willingness, not the actual willingness itself. Respond with a single number between 0 and 100, with one decimal place.

Separately, as part of exploratory diagnostic analyses, we conduct tests that remove or alter individual inputs (eg inverting first-order willingness, permuting country names, and reassigning GDP values) to assess whether LLM behavior is consistent with structured inference rather than memorization. Input wording and ordering were held constant across LLMs and countries.

### Models and decoding

We evaluate GPT-4o mini, Claude 3.5 Haiku, Gemini 2.5 Flash, and Llama 4 Maverick (17B) via their respective APIs over the period 2025 October—2026 January, following the public release of the GCCS paper (2024 February) and website (2024 April). Reported training data cutoffs, per publicly available documentation, are 2023 October (GPT-4o mini), 2024 July (Claude 3.5 Haiku), 2024 August (Llama 4 Maverick), and 2025 January (Gemini 2.5 Flash). Claude 3.5 Haiku was retired by Anthropic on 2026 February 19; the two-step prediction test conducted for this revision (2026 July) therefore used Claude Haiku 4.5 instead, benchmarked against the same model given real first-order input rather than against the original stage 5 results.

Temperature was fixed at zero to minimize sampling variation. However, hosted application programming interface (API) inference was not perfectly deterministic across separate runs. Outputs were parsed, retried where applicable, and bounded to the 0–100 interval using the analysis-specific procedures detailed in Supplementary Note S3.

### Evaluation and benchmarks

Model performance is assessed using two complementary metrics that capture different forms of predictive accuracy. MAE measures how close the predicted percentage is to the observed percentage in each country, and is therefore most relevant when the goal is to obtain an approximate estimate of the actual level of perceived support. Pearson correlation (*r*) measures whether the predictions capture cross-country variation. That is, whether countries predicted to have relatively high perceived support also tend to have relatively high observed support. A model can perform well on one metric but poorly on the other; for example, predictions concentrated near the global mean can have relatively low MAE while failing to distinguish meaningfully between countries. RMSE and variance explained (*R*^2^) are reported as secondary metrics.

For benchmarking, OLS and Lasso regressions are estimated using the same country-level covariates provided to the LLMs at stage 8, including first-order willingness. Statistical models are trained on 80% of countries and evaluated on a 20% holdout set, with repeated splits.

### Diagnostic tests

#### Sequential information addition

In preregistered experiments, we systematically varied the information provided to LLMs across eight stages: country name only (stage 1); adding sociodemographic variables (age, education, religiosity; stage 2); macroeconomic indicators (GDP per capita, top 1% income and wealth shares, HDI; stage 3); average temperature, 2010–2019 (stage 4); first-order willingness to act (stage 5); demographics plus macroeconomics combined (stage 6); adding temperature to stage 6 (stage 7); and the complete information set (stage 8). This design allows both MAE and correlation with ground truth to be tracked as information accumulates, and identifies which specific inputs drive changes in each.

#### Linear regressions of actual versus LLM-predicted outcomes

To examine country-level predictors of second-order beliefs, we estimated OLS regressions with robust standard errors, separately for (1) actual second-order beliefs and (2) each LLM's predicted second-order beliefs (Claude, GPT, Llama, Gemini). The dependent variable was the country-level mean of second-order beliefs (0–100 scale); explanatory variables were country-level means of first-order willingness, age, education, religiosity, HDI (2021), GDP per capita (2021), top 1% income share (2021), and average temperature (2010–2019).

This parallel modeling approach describes the correlational structure of each LLM's output relative to that of the actual data. It does not, on its own, establish that a model functionally relies on a given predictor, since covariates correlated with first-order willingness can appear as directionally correct predictors of LLM output even if a model does not use them directly; this distinction is addressed separately via the ablation and counterfactual tests below.

#### Feature ablations and counterfactuals

To understand how LLMs construct predictions, including which information they prioritize and whether they rely on memorized data, we removed individual inputs from the prompt containing all available information (stage 8) and separately changed selected inputs while holding the remaining information fixed. In exploratory analyses, we modified the preregistered prompt template by deleting one information block at a time while holding the remaining content fixed: all macroeconomic indicators (GDP per capita, top 1% income and wealth shares, HDI; no_econ); religion only (no_religion); demographics while retaining GDP per capita as a coarse development proxy (no_demo); temperature (no_climate); or all first-order willingness information (no_own_willingness). A country-name-only condition serves as a lower-bound benchmark.

Three counterfactual perturbations were also run. Personal willingness was flipped by replacing the willingness block with 75% (plus 15% additional) for baseline low-willingness countries (at or below 50%) and 15% (plus 10% additional) for high-willingness countries (above 50%), holding other inputs fixed (cf_willingness_flip). GDP per capita was flipped by replacing it with $65,000 for countries at or below $20,000 baseline and $2,000 for countries above that threshold, leaving other macroeconomic indicators unchanged (cf_gdp_flip). A name-data mismatch paired rich-country covariates (GDP per capita above $40,000) with poor-country names (GDP per capita below $5,000) and vice versa, updating the question text to reference the counterfactual name (cf_name_mismatch).

Even with identical preregistered prompts and temperature fixed at zero, predictions for nominally identical conditions (eg stage 1 vs the country-name-only ablation) were not always numerically identical across runs, reflecting small run-to-run variation in API outputs rather than substantive differences in inputs. This variation is small relative to ablation and counterfactual effects and does not affect the qualitative conclusions drawn.

### Tests against memorization

To assess whether main-study LLM performance reflects retrieval of country-specific survey values from training data rather than genuine use of the information provided, we administered three additional checks designed to detect memorization, specified in advance of testing but not part of the original preregistration (see Deviations). All tests used the same 125-country sample, ground-truth values, and prompting infrastructure as the main study, carried out as an exploratory analysis on 2026 April 17.

#### Direct recall probe (T1)

We constructed recall-inviting prompts naming the data source (3) and instructing LLMs to retrieve country-level second-order belief estimates from training data, the opposite of the retrieval-suppressing system prompt used in the main study. Predictions were elicited from all four LLMs (GPT-4o mini, Claude Haiku 4.5, Gemini 2.5 Flash, Llama 3.3 70B Instruct Turbo) and compared against stage 1 and stage 8 (retrieval suppressed) from the main study. Llama 3.3 70B substituted for Llama 4 Maverick, which was unavailable as a serverless endpoint at testing time; its December 2023 cutoff, predating GCCS publication, makes it a conservative substitute.

#### Training cutoff natural experiment (T2)

Under memorization, later training cutoffs, and thus greater postpublication exposure, should predict lower stage 8 MAE. We regressed each main-study LLM’s stage 8 MAE on its training cutoff date (GPT-4o mini: 2023 October; Claude 3.5 Haiku: 2024 July; Llama 4 Maverick: 2024 August; Gemini 2.5 Flash: 2025 January). With only four LLMs, this is a qualitative consistency check rather than a formal test.

#### Prepublication versus postpublication LLM comparison (T3)

We ran stage 8 prompts through definitively prepublication model versions, using the main study protocol. For Claude: Claude 3 Haiku (cutoff 2023 August) and Claude Haiku 4.5 (cutoff 2025 July) versus main-study Claude 3.5 Haiku (cutoff 2024 July). For Llama: Llama 3.3 70B (cutoff 2023 December, prepublication) versus main-study Llama 4 Maverick (cutoff 2024 August). MAE and Pearson *r* were computed for each version, with 95% bootstrap CIs (5,000 resamples).

### Robustness and replicability

We also repeated the analysis across 24 alternative second-order beliefs from three nationally representative US datasets (21, 23, 61), using prompts adapted to each specific policy (Supplementary Material, Note S2).

### Two-step prediction test

To test whether LLM-generated first-order estimates could substitute for real survey data in settings where none exist, we constructed a two-stage cascade using Claude Haiku 4.5 (see Models and decoding). In stage A, the model directly estimated each country's first-order willingness using only the sociodemographic, macroeconomic, and climate information available at stage 7, with no willingness data of any kind; the system prompt was adapted from the main study's second-order task to a first-order task (Supplementary Material, Note S1). In stage B, this predicted value, rather than the real first-order willingness, was used in a prompt with the same structure as stage 5 to generate second-order predictions.

To isolate the cost of cascading from differences between model generations, we benchmarked stage B against the same model (Claude Haiku 4.5) given the real first-order value in an otherwise identical stage 5 prompt (stage 5-real), rather than against the main study’s original stage 5 results, which used the now-retired Claude 3.5 Haiku. MAE, Pearson *r*, and Spearman rho were computed for stage A, stage B, and stage 5-real, with 95% bootstrap CIs (5,000 resamples); the stage B versus stage 5-real comparison used a paired bootstrap over the same resampled countries. This test was conducted on 2026 July 12.

### Deviations from preregistration

The study deviated from the preregistration in five respects. First, LLMs were evaluated across all *N* = 125 countries rather than the preregistered *n* = 112, because missing covariate values were explicitly indicated in prompts rather than used to exclude countries; results are robust to restricting the sample to complete-data countries (Figure S8 in the SI). Second, LLM predictions were evaluated on the full sample without train-test splits, since LLMs require no training data and out-of-sample evaluation does not apply; splits were retained for OLS and Lasso benchmarks. Third, the preregistration predicted prediction accuracy would increase monotonically with additional inputs, with the largest gain when mean willingness to contribute is added (Hypothesis 1); this held for Claude and Llama but not GPT and Gemini, which performed worse at stage 5 due to overweighting first-order willingness. Fourth, several reported analyses were not preregistered, including feature ablation tests, counterfactual perturbations, the three additional checks for memorization, structured-prompt robustness checks, and the 24-item US climate policy generalizability test; these are reported as exploratory throughout. Fifth, the two-step prediction test was conducted after the original study and preregistration, using Claude Haiku 4.5 rather than the main-study Claude 3.5 Haiku, which was retired by Anthropic between the original submission and this revision; it is reported as exploratory and benchmarked against the same model given real first-order input rather than against main-study stage 5 results, to avoid conflating the cost of cascading with differences between model generations.

## Supplementary Material

pgag374_Supplementary_Data

## Data Availability

Ground-truth second-order belief data and sociodemographic aggregates are derived from the Global Climate Change Survey administered as part of the Gallup World Poll 2021/2022 (3). Gallup World Poll microdata are subject to access restrictions and cannot be redistributed; researchers may apply for access at https://www.gallup.com/analytics/318923/world-poll-public-datasets.aspx. All country-level aggregated data, LLM predictions, and replication code are publicly available at https://github.com/npowdthavee/AIClimatePluralisticIgnorance. Macroeconomic and climate covariates are drawn from publicly available sources detailed in the Methods section and cited in the reference list below.

## AI usage

Artificial intelligence tools, including ChatGPT (OpenAI) and Grammarly, were used to polish language, enhance clarity, and organize content in the manuscript. No AI tools were used to produce scientific findings or perform analysis. All outputs were carefully reviewed and verified by the authors.

## Preprint version

An earlier version of this manuscript was posted on a preprint: https://arxiv.org/abs/2601.20141.

## Code and preregistration

Gallup World Poll microdata are subject to access restrictions, but all aggregation and analysis scripts are provided. The study was preregistered at https://osf.io/ez4ur/overview?view_only=e8add9f07f9148dfad359eed89744d5e.

## LLM reporting checklist

To support transparent reporting and reproducibility, the core details of the LLM runs are reported in the Methods section. Supplementary Note S3 provides additional model- and analysis-specific information, guided by the LLM reporting checklist for behavioral science, including exact API identifiers, parameter settings, prompt delivery formats, model substitutions, retry and parsing procedures, and software environment details (67).

## References

[1] Nielsen KS , et al 2024. Realizing the full potential of behavioural science for climate change mitigation. Nat Clim Chang. 14:322–330. 10.1038/s41558-024-01951-1

[2] Ostrom E . 2000. Collective action and the evolution of social norms. J Econ Perspect. 14:137–158.

[3] Andre P, Boneva T, Chopra F, Falk A. 2024. Globally representative evidence on the actual and perceived support for climate action. Nat Clim Chang. 14:253–259.

[4] Pörtner H-O , et al Summary for policymakers. Climate change 2022: impacts, adaptation and vulnerability. Contribution of working group II to the sixth assessment report of the intergovernmental panel on climate change. Cambridge University Press, 2022. p. 3–33.

[5] Climate Analytics & NewClimate Institute . 2019. Climate Action Tracker. https://climateactiontracker.org/.

[6] Bergquist M, Nilsson A, Schultz WP. 2019. A meta-analysis of field-experiments using social norms to promote pro-environmental behaviors. Glob Environ Change. 59:101941.

[7] Boon-Falleur M, Grandin A, Baumard N, Chevallier C. 2022. Leveraging social cognition to promote effective climate change mitigation. Nat Clim Change. 12:332–338.

[8] Cialdini RB, Reno RR, Kallgren CA. 1990. A focus theory of normative conduct: Recycling the concept of norms to reduce littering in public places. J Pers Soc Psychol. 58:1015.

[9] Jachimowicz JM, Hauser OP, O’Brien JD, Sherman E, Galinsky AD. 2018. The critical role of second-order normative beliefs in predicting energy conservation. Nat Hum Behav. 2:757–764. 10.1038/s41562-018-0434-0 31406290

[10] Mildenberger M, Tingley D. 2019. Beliefs about climate beliefs: the importance of second-order opinions for climate politics. Brit J Polit Sci. 49:1279–1307.

[11] Tankard ME, Paluck EL. 2016. Norm perception as a vehicle for social change. Soc Issues Policy Rev. 10:181–211.

[12] Bicchieri C . Norms, conventions, and the power of expectations. Philosophy of social sciences: a new introduction. Oxford University Press, 2014. p. 208–229.

[13] Korte C . 1972. Pluralistic ignorance about student radicalism. Sociometry. 35:576–587.

[14] O’Gorman H . 1986. The discovery of pluralistic ignorance: an ironic lesson. J Hist Behav Sci. 22:333–347.

[15] Prentice DA, Miller DT. 1993. Pluralistic ignorance and alcohol use on campus: some consequences of misperceiving the social norm. J Pers Soc Psychol. 64:243–256. 10.1037//0022-3514.64.2.243 8433272

[16] Leviston Z, Walker I, Morwinski S. 2013. Your opinion on climate change might not be as common as you think. Nat Clim Change. 3:334–337.

[17] Geiger SJ , et al 2025. What we think others think and do about climate change: a multicountry test of pluralistic ignorance and public-consensus messaging. Psychol Sci. 36:421–442. 10.1177/09567976251335585 PMC13428961 40403301

[18] Ban Rohring EJ, Akerlof KL. 2020. Perceptions of social consensus at the regional level relate to prioritization and support of climate policy in Maryland, USA. Reg Environ Change. 20:1–13.

[19] Geiger N, Swim JK. 2016. Climate of silence: pluralistic ignorance as a barrier to climate change discussion. J Environ Psychol. 47:79–90.

[20] Leviston Z, Nangrani T, Stanley SK, Walker I. 2024. Consequences of group-based misperceptions of climate concern for efficacy and action. Curr Res Ecol Soc Psychol. 6:100189.

[21] Sparkman G, Geiger N, Weber EU. 2022. Americans experience a false social reality by underestimating popular climate policy support by nearly half. Nat Commun. 13:4779. 10.1038/s41467-022-32412-y PMC9399177 35999211

[22] Dixon G, Clarke C, Jacquet J, Evensen DTN, Hart PS. 2024. The complexity of pluralistic ignorance in Republican climate change policy support in the United States. Commun Earth Environ. 5:1–6.

[23] Lees J, Colaizzi G, Goldberg MH, Constantino SM. Misperceptions of support for climate policy represent multiple phenomena predicted by different factors across intergroup boundaries. 2023. 10.31219/osf.io/vfbq4

[24] Poortinga W . 2026. The role of policy appraisals and second-order beliefs in public support for climate policies in the UK. Clim Policy. 26:1215–1229. 10.1080/14693062.2025.2539977

[25] Van Boven L, Ehret PJ, Sherman DK. 2018. Psychological barriers to bipartisan public support for climate policy. Perspect Psychol Sci. 13:492–507. 10.1177/1745691617748966 29961412

[26] Cole JC, Ehret PJ, Sherman DK, Van Boven L. 2022. Social norms explain prioritization of climate policy. Clim Change. 173:10. 10.1007/s10584-022-03396-x PMC9289929 35874038

[27] Tiede KE, Maur K, Betsch C. 2026. People systematically under- and overestimate public engagement in climate action. Nat Clim Chang. 16:774–780.

[28] Chen S, Wan F, Yang S. 2022. Normative misperceptions regarding pro-environmental behavior: mediating roles of outcome efficacy and problem awareness. J Environ Psychol. 84:101917.

[29] Andre P, Boneva T, Chopra F, Falk A. 2026. Misperceived social norms and willingness to act against climate change. Rev Econ Stat. 108:1205–1221. 10.1162/rest_a_01468

[30] Geiger SJ , et al 2025. Understanding the role of pluralistic ignorance in biodiversity conservation: a research agenda. Glob Environ Change. 95:103043.

[31] Fischbacher U, Gächter S, Fehr E. 2001. Are people conditionally cooperative? Evidence from a public goods experiment. Econ Lett. 71:397–404.

[32] Fehr E, Fischbacher U. 2004. Social norms and human cooperation. Trends Cogn Sci. 8:185–190. 10.1016/j.tics.2004.02.007 15050515

[33] Rustagi D, Engel S, Kosfeld M. 2010. Conditional cooperation and costly monitoring explain success in forest commons management. Science. 330:961–965. 10.1126/science.1193649 21071668

[34] Orr M , et al 2025. Breaking the climate silence: predictors of discussing global warming with family and friends. PLOS Clim. 4:e0000538.

[35] Bouman T, van der Werff E, Perlaviciute G, Steg L. 2021. Environmental values and identities at the personal and group level. Curr Opin Behav Sci. 42:47–53.

[36] Bouman T, Steg L. 2022. A spiral of (in)action: empowering people to translate their values in climate action. One Earth. 5:975–978.

[37] Noelle-Neumann E . The spiral of silence: public opinion–our social skin. University of Chicago Press, 1993.

[38] de Groot JIM, Schuitema G. 2012. How to make the unpopular popular? Policy characteristics, social norms and the acceptability of environmental policies. Environ Sci Policy. 19–20:100–107.

[39] Vlasceanu M , et al 2024. Addressing climate change with behavioral science: a global intervention tournament in 63 countries. Sci Adv. 10:eadj5778. 10.1126/sciadv.adj5778 PMC10849597 38324680

[40] Drews S, Savin I, van den Bergh JCJM. 2022. Biased perceptions of other people's attitudes to carbon taxation. Energy Policy. 167:113051.

[41] Kajackaite A, Galdikiene L, Jaraite J. 2025. Pluralistic ignorance and climate policies: information provision experiment [preprint]. *SSRN Scholarly*, 1–61. 10.2139/ssrn.5265851

[42] Bursztyn L, Yang DY. 2022. Misperceptions about others. Annu Rev Econ. 14:425–452.

[43] Lees J, Cikara M. 2020. Inaccurate group meta-perceptions drive negative out-group attributions in competitive contexts. Nat Human Behav. 4:279–286. 10.1038/s41562-019-0766-4 31712763

[44] Većkalov B , et al 2024. A 27-country test of communicating the scientific consensus on climate change. Nat Human Behav. 8:1892–1905. 10.1038/s41562-024-01928-2 PMC11493676 39187712

[45] Pataranutaporn P, Powdthavee N, Archiwaranguprok C, Maes P. 2025. Simulating human well-being with large language models: systematic validation and misestimation across 64,000 individuals from 64 countries. Proc Natl Acad Sci U S A. 122:e2519394122. 10.1073/pnas.2519394122 PMC12685116 41296717

[46] Argyle LP , et al 2023. Out of one, many: using language models to simulate human samples. Political Anal. 31:337–351.

[47] Qu Y, Wang J. 2024. Performance and biases of large language models in public opinion simulation. Humanit Soc Sci Commun. 11:1095.

[48] Suh J, Jahanparast E, Moon S, Kang M, Chang S. 2025. Language model fine-tuning on scaled survey data for predicting distributions of public opinions. [preprint], arXiv:2502.16761, 10.48550/arXiv.2502.16761

[49] Lee K, Park J, Choi S, Lee C. 2025. Ideology and policy preferences in synthetic data: the potential of LLMs for public opinion analysis. Media Commun. 13:1–20. 10.17645/mac.9677

[50] Jiang S, Wei L, Zhang C. 2024. Donald trumps in the virtual polls: Simulating and predicting public opinions in surveys using large language models [preprint], arXiv:2411.01582v2, https://arxiv.org/abs/2411.01582v2.

[51] von der Heyde L, Haensch, A-C, Wenz, A. 2024. Vox populi, vox AI? Using language models to estimate German public opinion [preprint], arXiv:2407.08563v1.

[52] Lee S , et al 2024. Can large language models estimate public opinion about global warming? An empirical assessment of algorithmic fidelity and bias. PLoS Clim. 3:e0000429.

[53] Xia Z , et al 2025. Predicting climate policy support with large language models: assessing accuracy and bias in AI-simulated policy support. SSRN Scholarly. 10.2139/ssrn.5266427

[54] Wang A, Morgenstern J, Dickerson JP. 2025. Large language models that replace human participants can harmfully misportray and flatten identity groups. Nat Mach Intell. 7:400–411.

[55] Marchetti A, Manzi F, Riva G, Gaggioli A, Massaro D. 2025. Artificial intelligence and the illusion of understanding: a systematic review of theory of mind and large language models. Cyberpsychol Behav Soc Netw. 28:505–514. 10.1089/cyber.2024.0536 40333375

[56] Galitsky BA . Improving ToM capabilities of LLMs in applied domains. 2025. 10.20944/preprints202502.1456.v1

[57] Miao Y, Zhu J, Shwartz V. 2026. LLMs infer cultural context but fail to apply it when responding [preprint], arXiv:2606.17688, https://arxiv.org/abs/2606.17688.

[58] Robbins JM, Krueger JI. 2005. Social projection to ingroups and outgroups: a review and meta-analysis. Pers Soc Psychol Rev. 9:32–47. 10.1207/s15327957pspr0901_3 15745863

[59] Messeri L, Crockett MJ. 2024. Artificial intelligence and illusions of understanding in scientific research. Nature. 627:49–58. 10.1038/s41586-024-07146-0 38448693

[60] Abdurahman S , et al 2024. Perils and opportunities in using large language models in psychological research. PNAS Nexus. 3:245. 10.1093/pnasnexus/pgae245 PMC11249969 39015547

[61] Swim J, Baker Z. Americans realistically assess public views of climate change as a national problem. 2025. 10.21203/rs.3.rs-7633595/v1

[62] Zewail A, Figueroa A, Graham J, Atari M. 2026. Moral stereotyping in large language models. Proc Natl Acad Sci U S A. 123:e2519941123. 10.1073/pnas.2519941123 PMC12974530 41779787

[63] Feuerriegel S, Maarouf A, Demir S, Weinmann M. 2025. Analyzing heterogeneity in the effects of behavioral climate interventions on individual beliefs across sociodemographics, attitudes, and norms. Research Square. Preprint at 10.21203/rs.3.rs-7519080/v1

[64] World Bank . GDP Per capita (current international $). World Bank Open Data, 2024. https://data.worldbank.org/indicator/NY.GDP.PCAP.PP.CD.

[65] Alvaredo F, Atkinson AB, Piketty T, Saez E. WID.world. World Inequality Database. 2024. [accessed 2025 Oct 28]. https://wid.world/data

[66] World Bank . Human development index (HDI). 2024. [accessed 2025 Oct 28]. https://databank.worldbank.org/Human-development-index/id/363d401b

[67] Feuerriegel S, et al 2026. A reporting checklist for large language models in behavioural science. Nat Hum Behav. 10:1182–1186. 10.1038/s41562-026-02492-7 42265331

