# Supplementary material for: Can large language models predict public perceptions of support for climate action across 125 countries?
Source: PNAS Nexus. 2026 Oct 31;5(11):pgag374. doi: 10.1093/pnasnexus/pgag374 (PMC13755878; doi:10.1093/pnasnexus/pgag374)
Supplement: Supplementary file 1 [file pgag374_supplementary_data.pdf]

Supplementary Materials

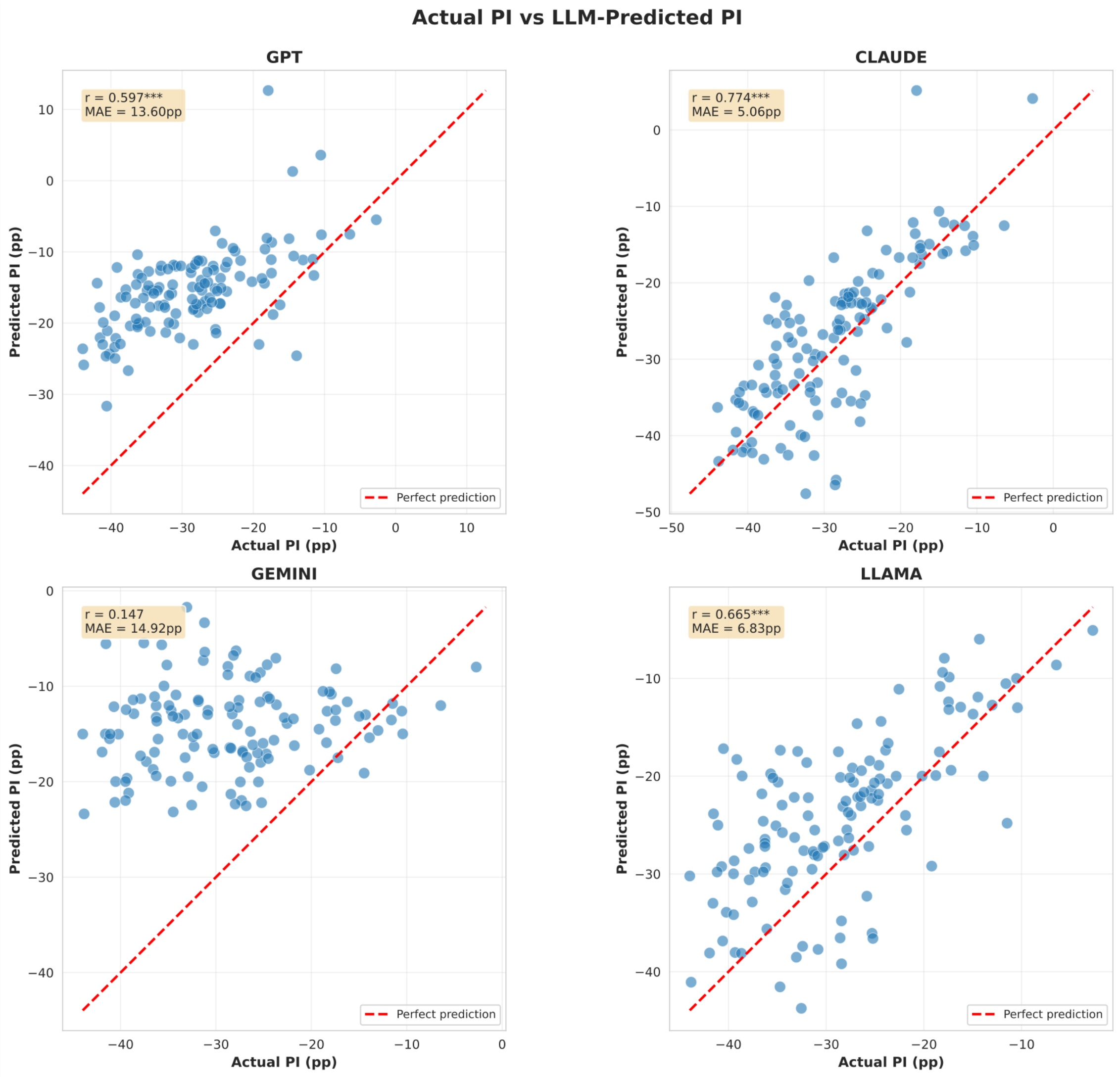

Fig. S1. Country-level scatterplots comparing ground truth to LLM-predicted second-order beliefs (perceived others' willingness to contribute, 0-100%).

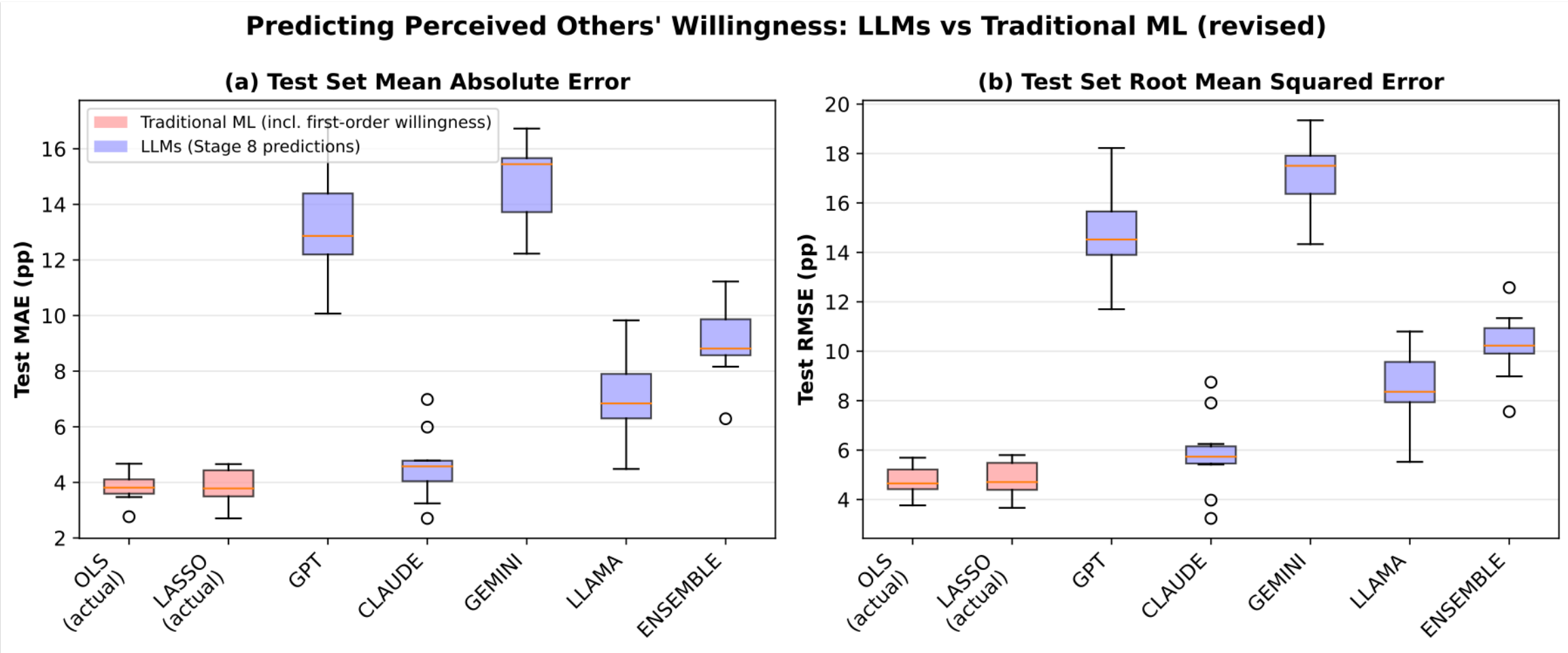

Fig. S2. Performance benchmarking for predicting perceptions of others' willingness to contribute financially to address climate change across LLMs: (*a*) test-set mean absolute error (MAE) and (*b*) test-set root mean square error (RMSE). Both panels restrict all methods, including the fixed LLM predictions, to the same complete-case sample of countries. In each of ten repeated splits, OLS and Lasso were trained on 80% of countries, and all methods were evaluated on the same 20% holdout countries.

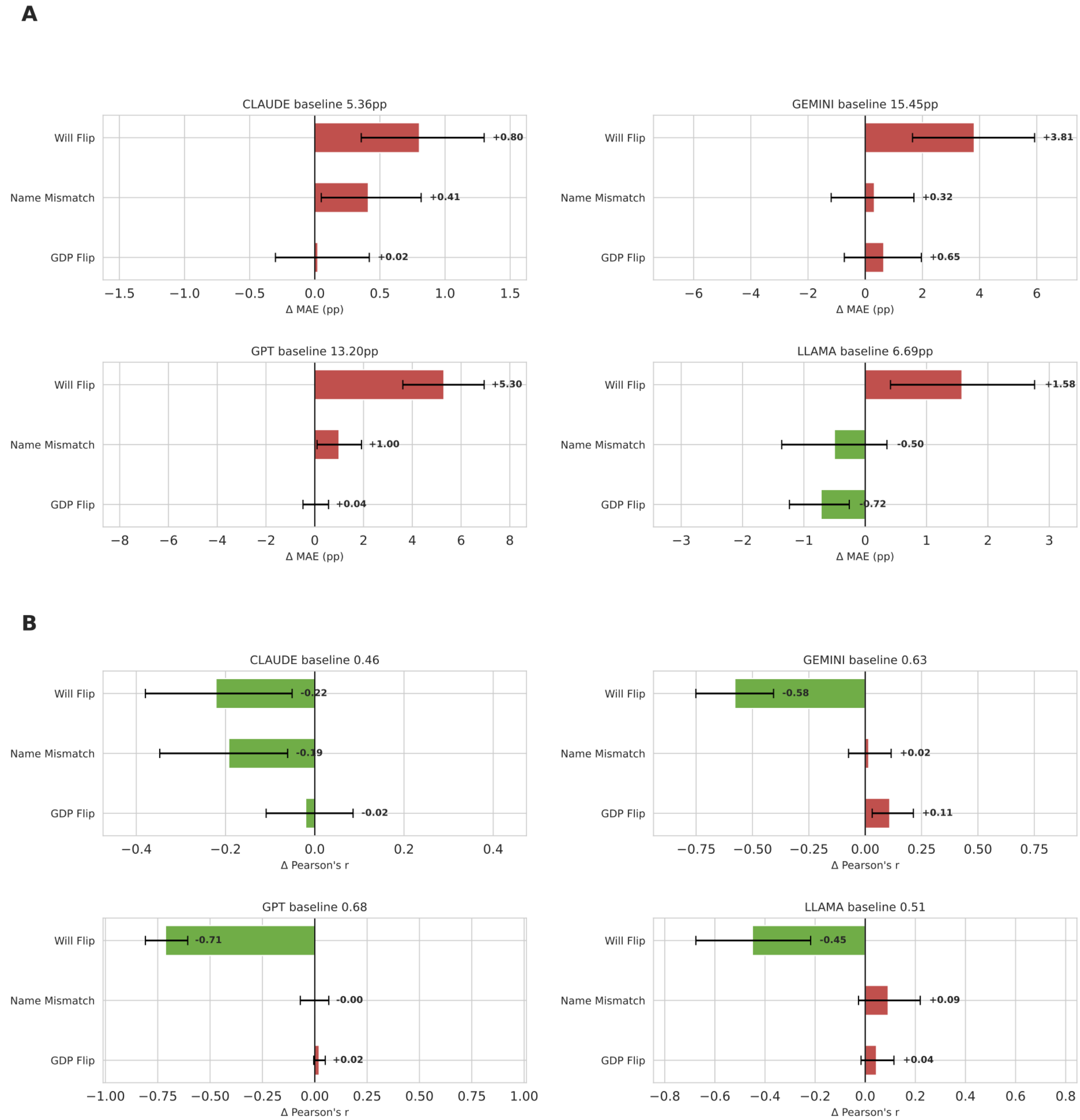

Fig. S3. Counterfactual robustness tests. Panel (A) - Change in MAE relative to the full specification under three counterfactual perturbations: willingness-flip (personal willingness randomized across countries), name-mismatch (country labels reassigned while other attributes are held constant), and GDP-flip (economic values randomized across countries). Panel (B) - Corresponding change in Pearson's r for the same perturbations. Error bars show 95% CI. A perturbation that substantially changes both MAE and correlation provides evidence that predictions depend on that input. Stability under a perturbation provides no evidence of a meaningful effect under the tested perturbation.

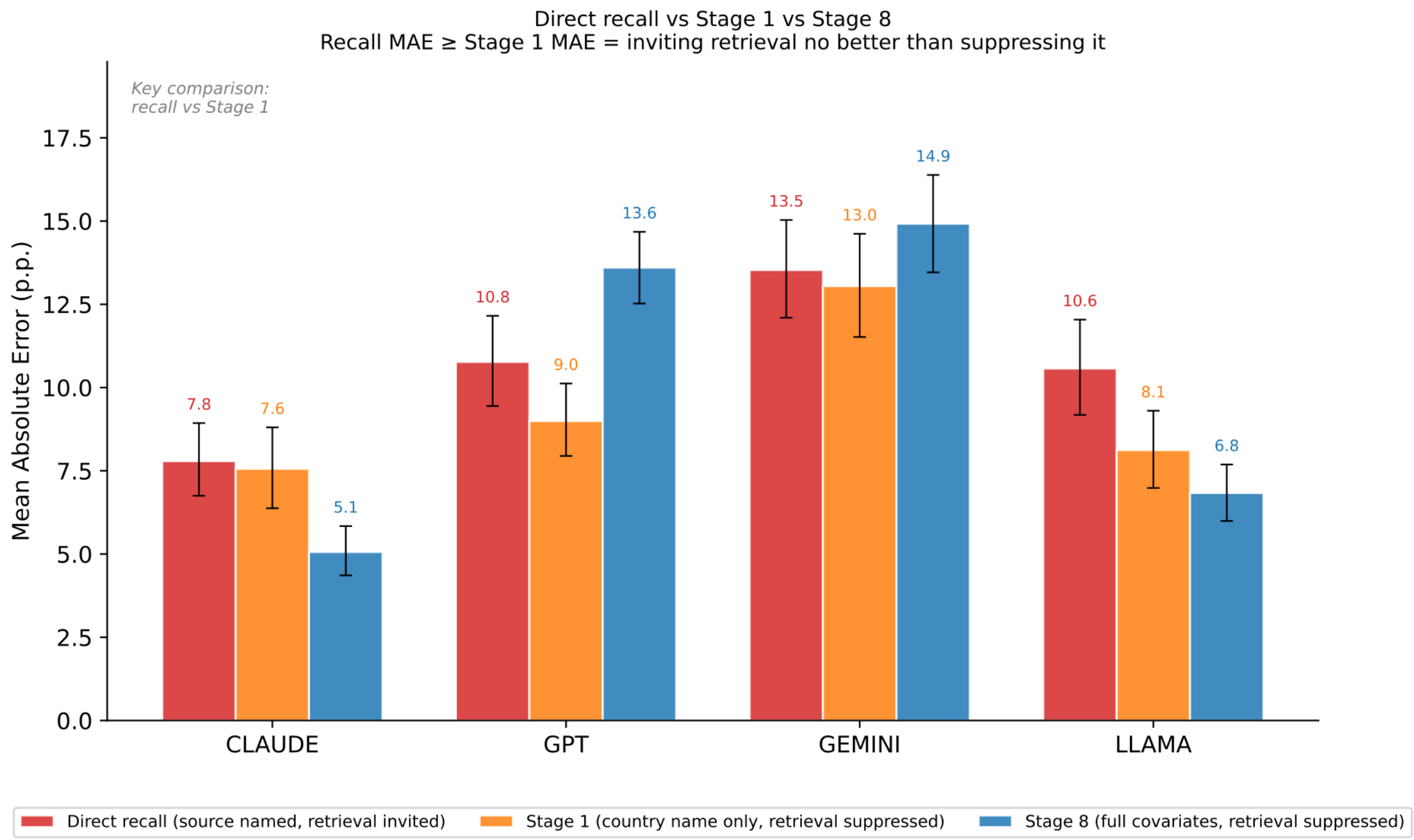

Fig. S4. Direct recall probe: Comparing retrieval-invited vs retrieval-suppressed predictions. Grouped bar chart showing mean absolute error (MAE, p.p.) for each LLM under three conditions: direct recall (red; prompts naming Andre et al., 2024, *Nature Climate Change* as the source, with system instructions actively inviting retrieval from training data), Stage 1 (orange; country name only, retrieval suppressed), and Stage 8 (blue; full covariate information, retrieval suppressed). Error bars represent 95% bootstrap confidence intervals (10,000 resamples). The key comparison is between direct recall and Stage 1 — both provide minimal country-level information, but recall actively invites retrieval while Stage 1 suppresses it. For Claude and Llama, direct-recall MAE exceeds Stage 1 MAE, indicating that explicitly invoking retrieval yields no improvement over suppressing it. These results are inconsistent with the view that country-specific memorization of GCCS values is the primary driver of performance in the main study. Note: Llama 4 Maverick (main study) was unavailable as a serverless endpoint at the time of robustness testing and was replaced with Llama 3.3 70B Instruct Turbo (training cutoff December 2023, pre-GCCS publication), making the recall test conservative — a pre-publication LLM cannot retrieve values it has never been exposed to.

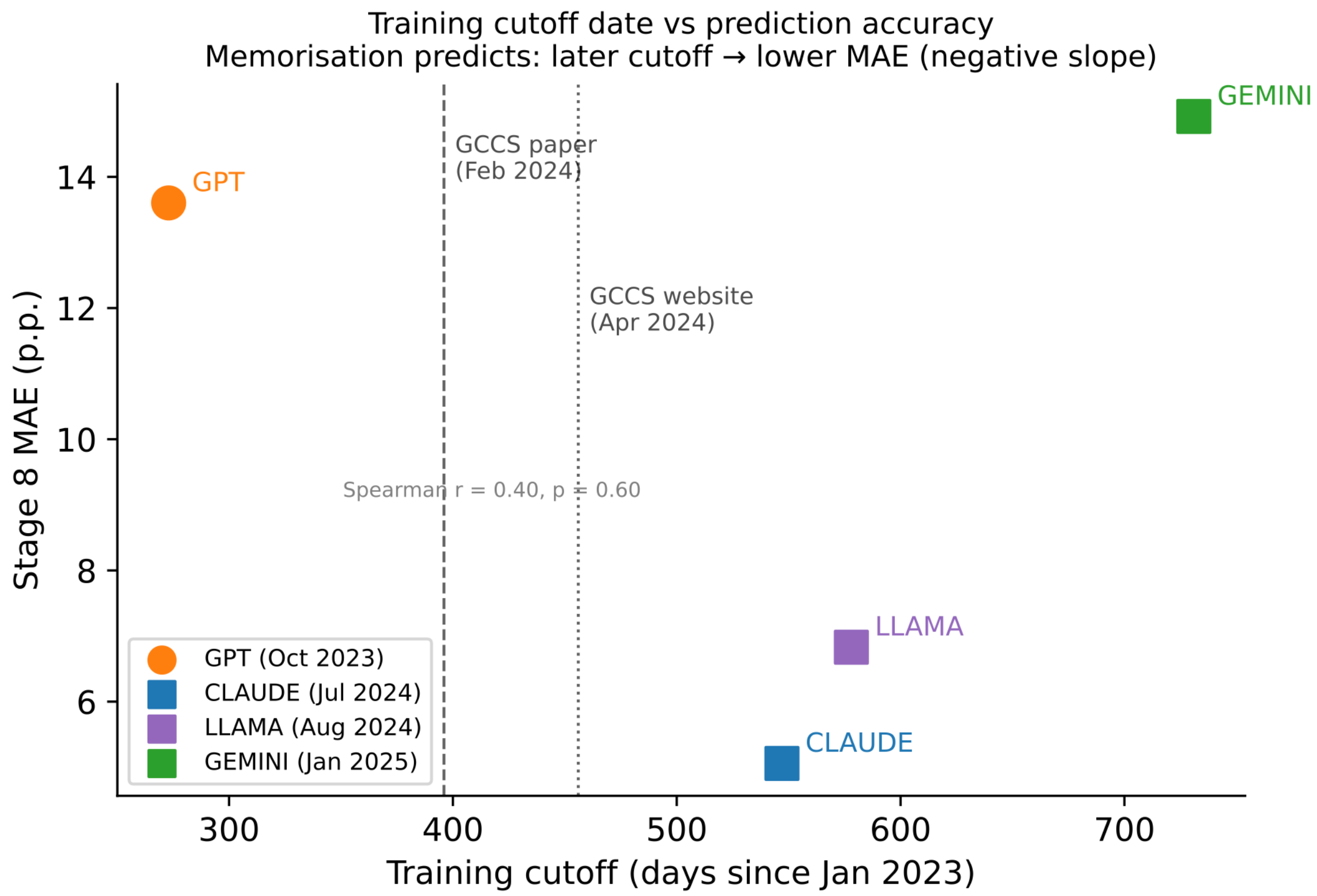

Fig. S5. Training cutoff natural experiment. Scatter plot showing the relationship between each "main study" LLM's training data cutoff (days since January 2023, x-axis) and Stage 8 MAE (p.p., y-axis). Circle markers indicate pre-GCCS LLMs (GPT-4o mini, cutoff October 2023); square markers indicate post-GCCS LLMs (Claude 3.5 Haiku, July 2024; Llama 4 Maverick, August 2024; Gemini 2.5 Flash, January 2025). Vertical dashed and dotted lines mark GCCS paper publication (February 2024) and GCCS website launch (April 2024), respectively. If memorization drives performance, later cutoffs should yield lower MAEs (i.e., a negative slope). The absence of a negative cutoff–MAE relationship is inconsistent with post-publication data exposure as the primary driver of main-study performance.

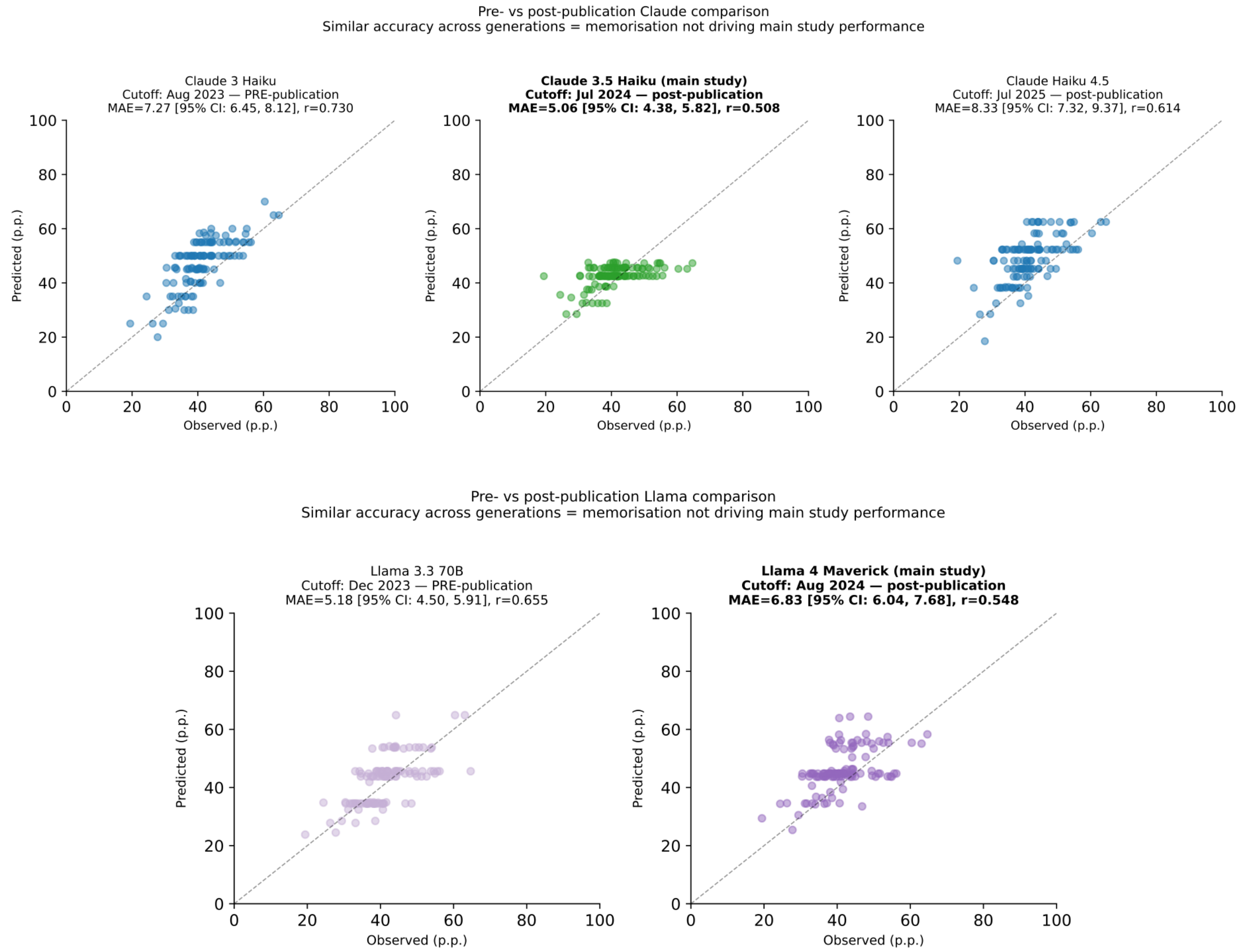

Fig. S6. Pre- vs post-publication LLM comparison. Scatter plots showing observed vs predicted second-order beliefs (p.p.) across 125 countries for pre- and post-publication LLM versions, using Stage 8 prompts and the original main study system prompt. The dashed diagonal represents perfect prediction. **Panel A — Claude** (top row): Claude 3 Haiku (cutoff August 2023, PRE-publication; MAE = 7.27 p.p., r = 0.730), Claude 3.5 Haiku (cutoff July 2024, post-publication; MAE = 5.06 p.p., r = 0.508; main study LLM, bold), and Claude Haiku 4.5 (cutoff July 2025, post-publication; MAE = 8.33 p.p., r = 0.614). **Panel B — Llama** (bottom row): Llama 3.3 70B (cutoff December 2023, PRE-publication; MAE = 5.18 p.p. [95% CI: 4.50–5.91], r = 0.655) and Llama 4 Maverick (cutoff August 2024, post-publication; MAE = 6.83 p.p. [95% CI: 6.04–7.68], r = 0.548; main study LLM, bold). In both panels, pre-publication LLMs perform comparably to or better than their post-publication counterparts, providing no evidence that exposure to GCCS data after publication drives main-study performance.

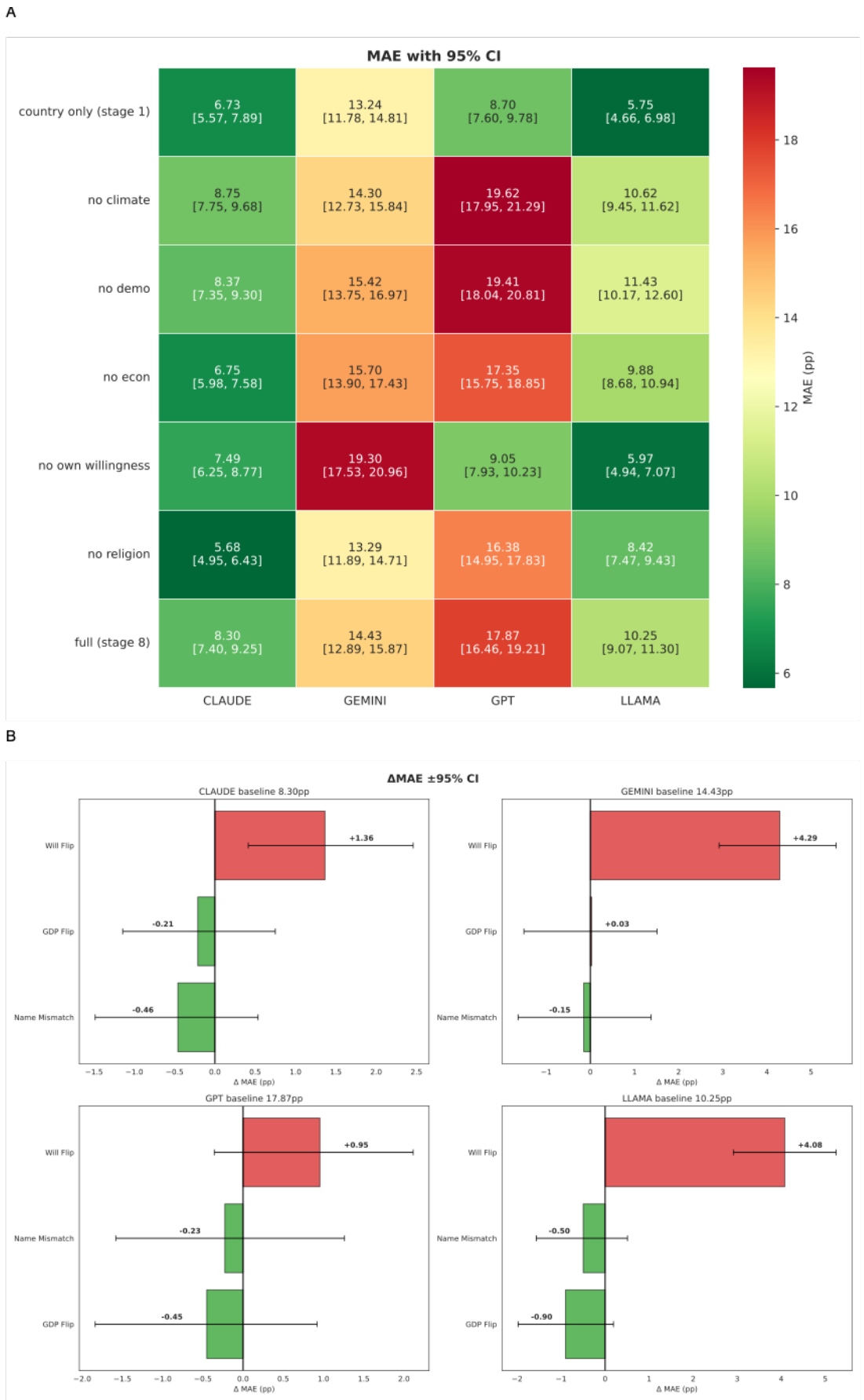

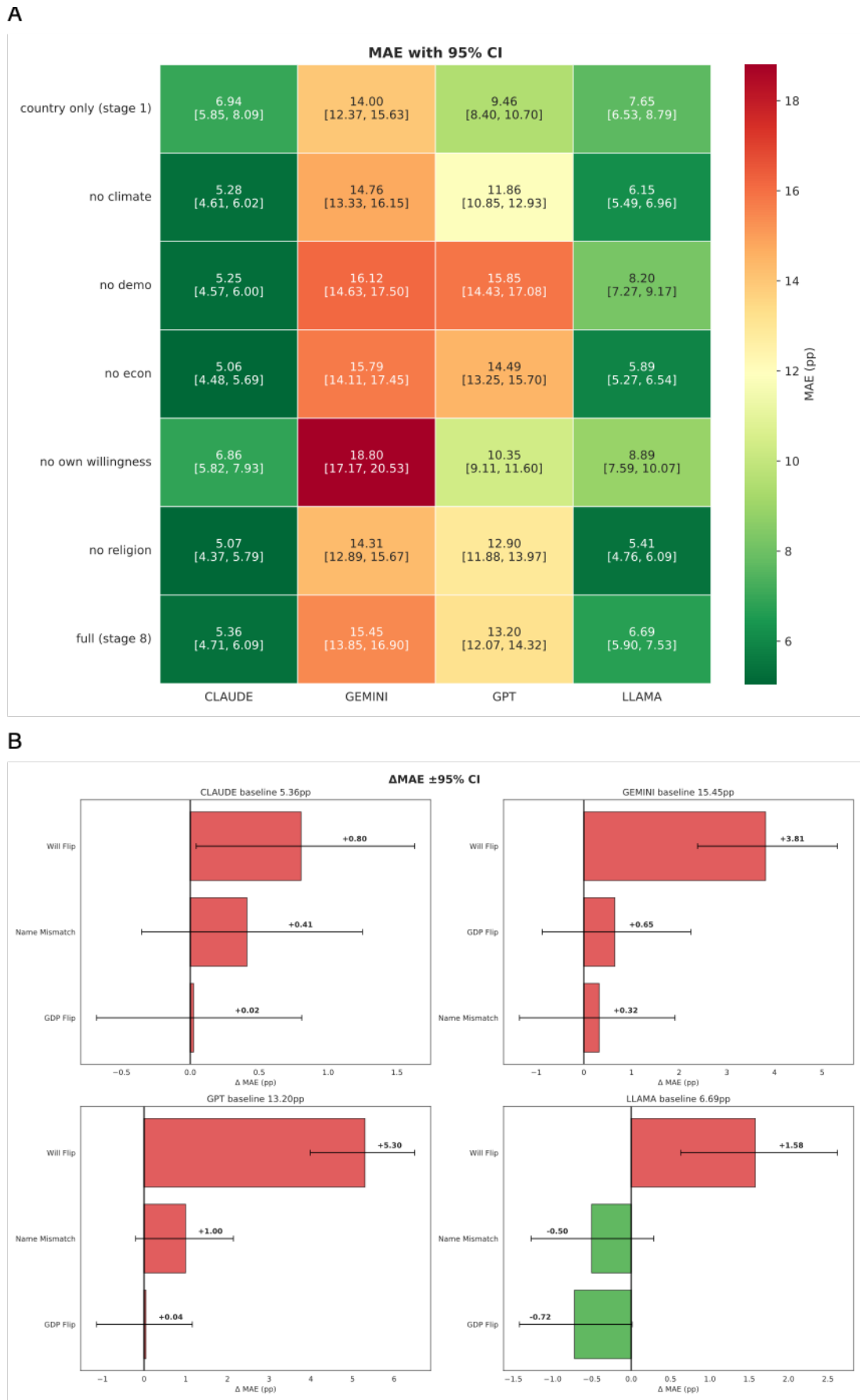

i) Structured prompting

ii) Natural language prompting

Fig. S7: Robustness to prompt format: Structured vs. natural language presentation. Comparison of LLM performance using structured (bullet-point) versus natural-language prompts. Panels show mean absolute error (MAE) for ablation tests and counterfactual manipulations under both prompt formats. Country attributes (demographics, GDP, climate indicators, first-order beliefs) were identical across formats; only presentation style varied. Results demonstrate that core findings—particularly the importance of first-order willingness and robustness to GDP/name swaps—replicate across prompt structures.

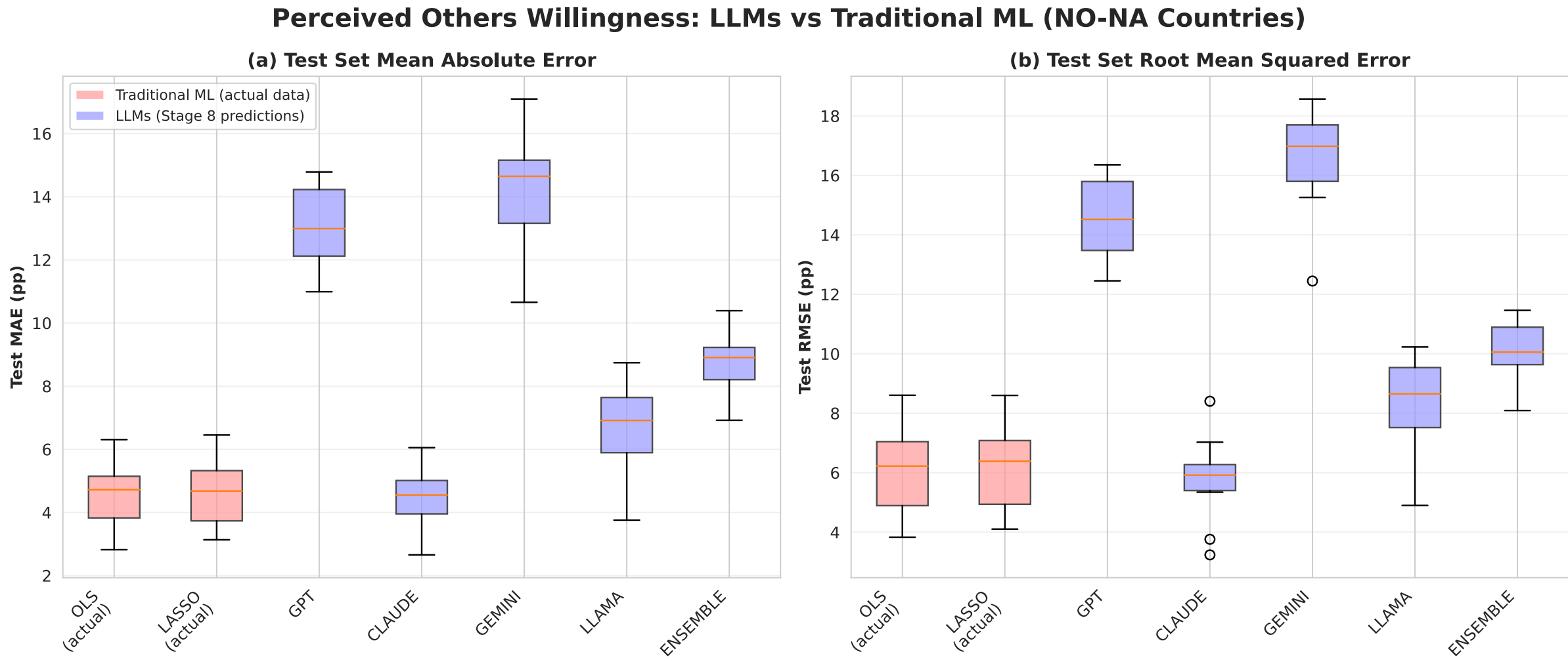

Fig. S8: LLM Performance vs. traditional machine learning (ML) on complete-data sample. Comparison of LLM predictions (stage 8) against ordinary least squares (OLS) and LASSO regression trained on actual country-level data, restricted to 112 countries with complete information for all variables (excluding 13 countries with any missing values). Statistical models were evaluated using 10 repeated 80:20 train-test splits stratified by continent. (a) Test set mean absolute error (MAE). (b) Test set root mean squared error (RMSE). Boxplots show distribution across 10 iterations. Traditional ML models (red) are trained on actual data; LLMs (blue) rely on prompted inference without training.

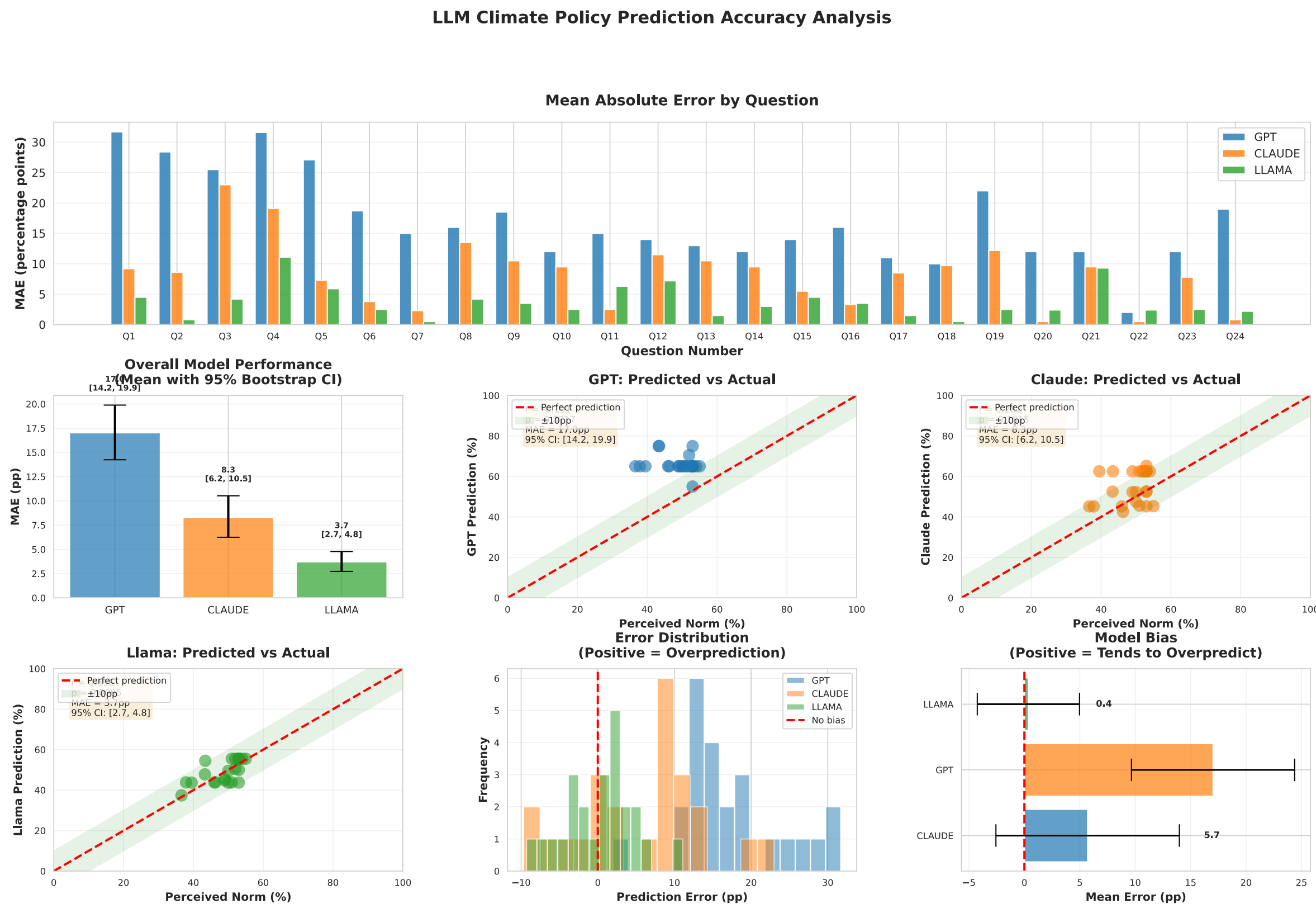

Fig. S9: Generalization to U.S. climate policy (24 items). LLM performance predicting public perceptions of others' support across 24 climate policy items from three nationally representative U.S. datasets[21,23,64]. Top panel: Mean absolute error (MAE) by question for GPT, Claude, and Llama across all 24 items. Middle left: Overall LLM performance (mean with 95% bootstrap CI). Middle center and right: Predicted vs. actual scatter plots for GPT and Claude showing correlations with ground truth. Bottom left: Predicted vs. actual for Llama. Bottom center: Error distribution across all predictions, showing frequency of overestimation vs. underestimation. Bottom right: Model bias (mean error), with positive values indicating overestimation and negative values indicating underestimation. Gemini produced no predictions for any items, likely due to content filtering of policy-related queries.

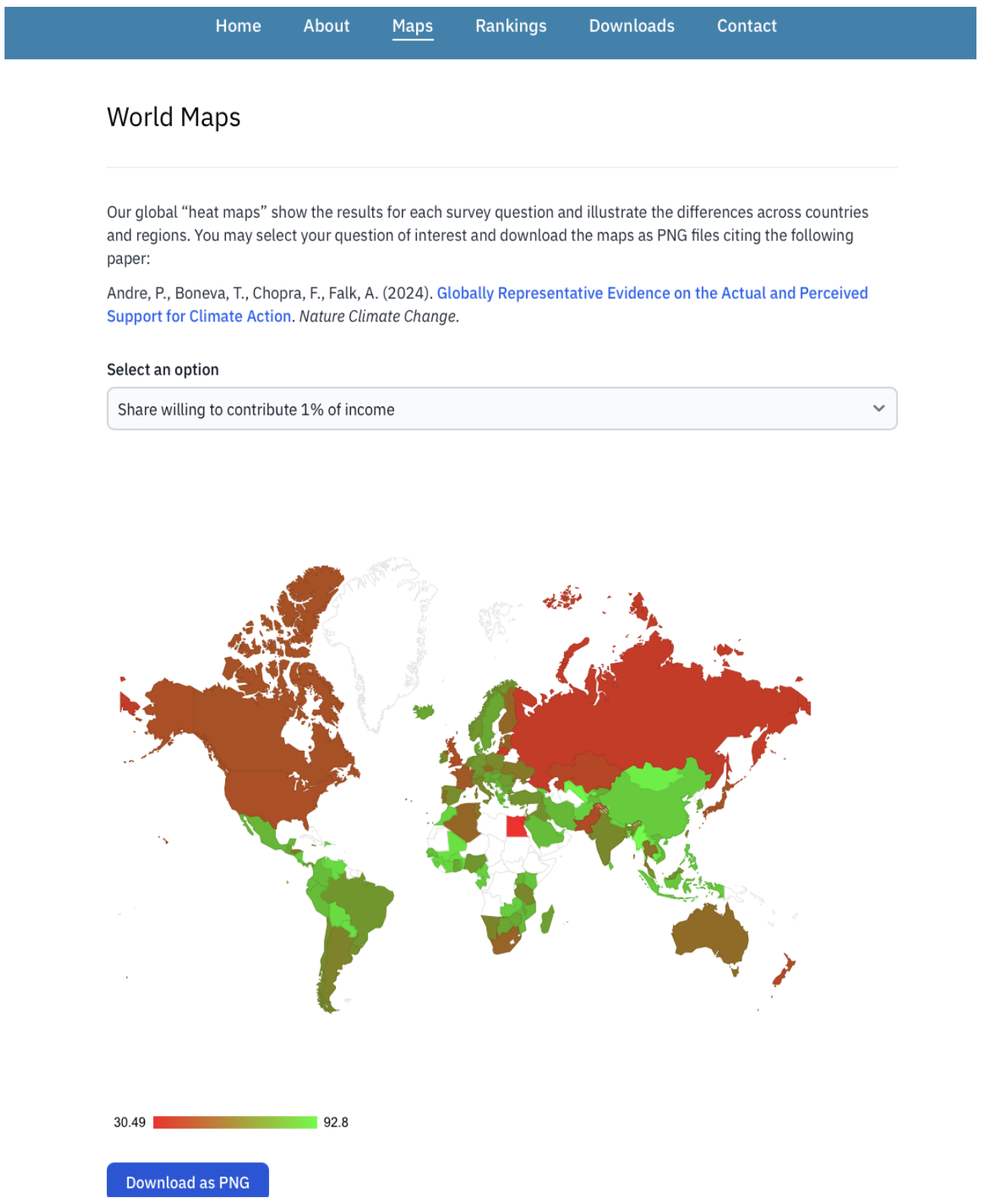

a) Map page

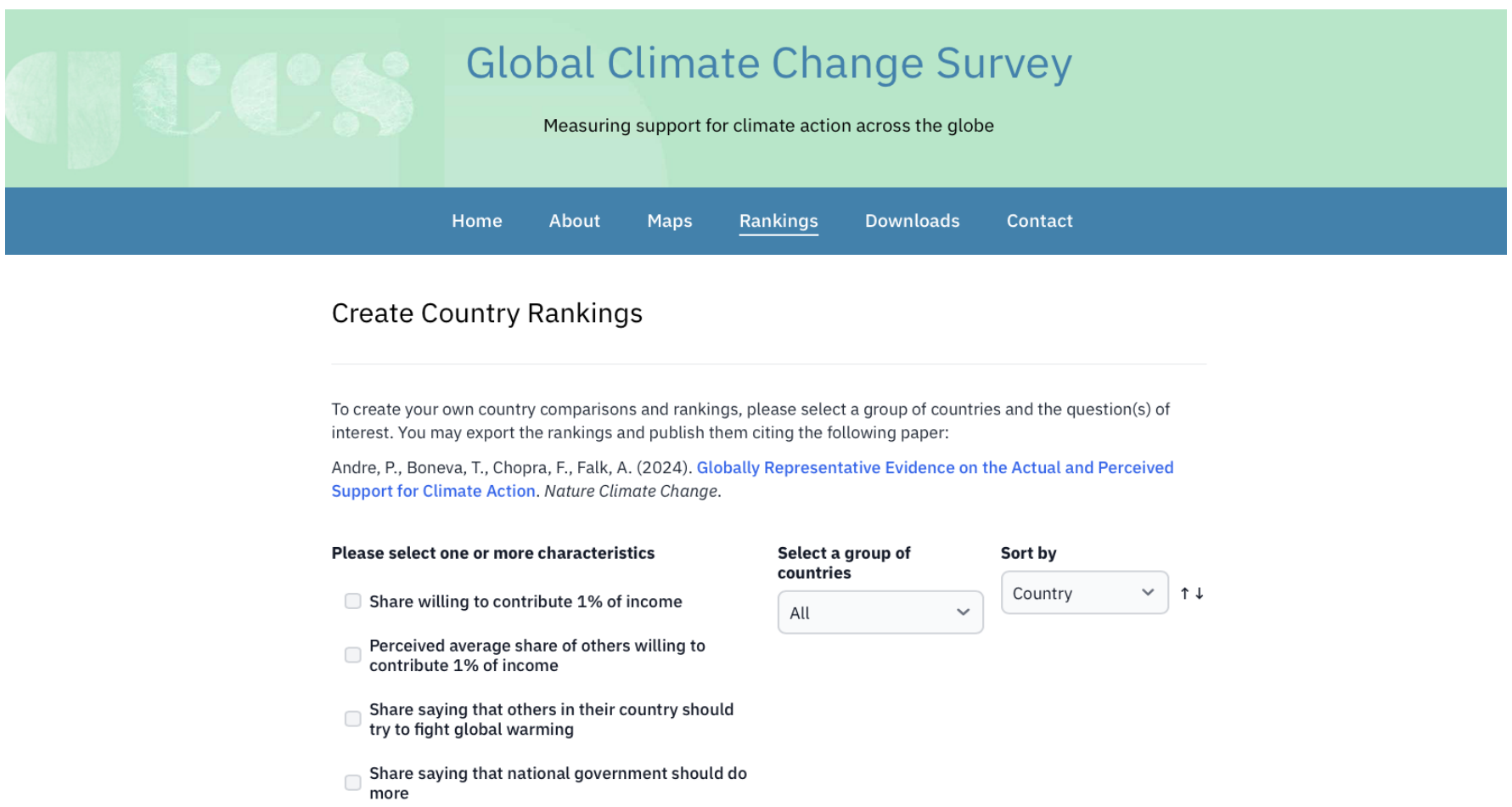

b) Rankings page

Fig. S10: GCCS website interactive data access interfaces. (a) Maps page (https://gccs-survey.org/maps): Interactive heat map showing second-order beliefs about willingness to contribute 1% of income toward climate action. Country-level values are revealed only through individual hover interactions. The color gradient (30.49-92.8% range shown) provides relative comparisons but not precise numerical values. (b) Rankings page (https://gccs-survey.org/rankings): Custom ranking generator requiring users to select survey questions, country groups, and sorting criteria before generating exportable country comparisons. No pre-generated rankings or comprehensive datasets are available without active user configuration. Both interfaces require interactive engagement to access country-specific data, limiting the availability of systematic country-level rankings during potential LLM training.

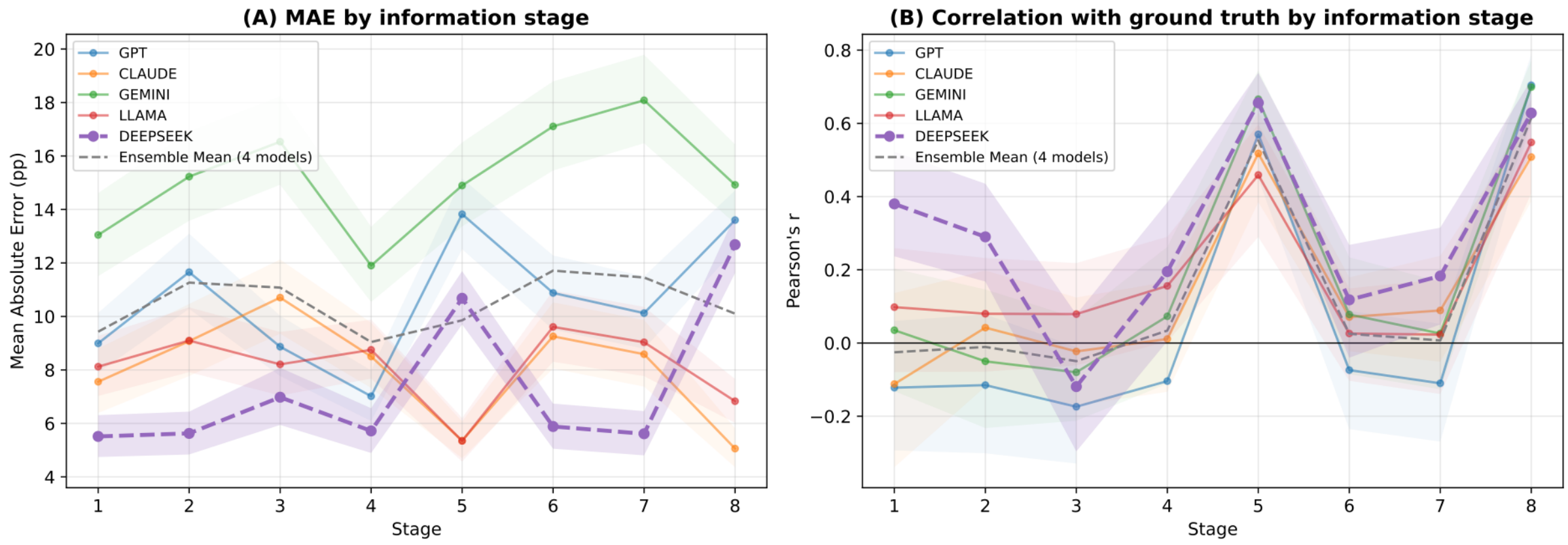

Fig. S11: Robustness to model training origin. Staged information-addition results (Stages 1–8) for DeepSeek-V3.1, a Chinese-developed model, plotted against the four main-study models (GPT, Claude, Gemini, Llama) and their ensemble mean. (A) Mean absolute error (MAE, p.p.) by information stage. (B) Pearson's r with ground truth by information stage. Shaded bands represent 95% bootstrap confidence intervals (10,000 resamples). At Stage 8, DeepSeek (MAE = 12.68 p.p. [11.63, 13.77], r = .628 [.519, .719]) shows a degradation pattern once first-order willingness is introduced (Stages 5, 8) similar to GPT and Gemini, but tracks country rankings more closely than Claude or Llama at full information. DeepSeek also showed above-chance correlation at Stage 1 (country name only; r = .380 [.237, .522]), not observed for any other model; a direct recall probe (see Direct recall probe, Methods) performed worse than both Stage 1 and Stage 8 on error (MAE = 16.78 p.p. [15.33, 18.22]) while showing comparable correlation (r = .349 [.188, .504]), arguing against memorization of country-specific values as the source of the Stage 1 result. This is a single exploratory model addition, not a systematic test of training-origin effects.

Note 1: System Instruction for LLM Queries

To prevent data leakage and ensure that predictions rely on inference rather than retrieval of memorized survey results, all LLM queries included the following system instruction:

"You are a prediction assistant making estimates based ONLY on the information provided in this specific prompt.

CRITICAL INSTRUCTIONS:

1. Do NOT cite, reference, or mention ANY research papers, academic studies, surveys, or authors (including but not limited to André et al., Sparkman et al., Leviston et al., Lees et al., or any other researchers).
2. Do NOT use any memorized data, statistics, or percentages from your training about climate change opinions, pluralistic ignorance, or survey results.
3. Treat this as a completely NOVEL scenario—ignore any similar studies you may have seen during training.
4. Do NOT reference phrases such as 'research shows,' 'studies indicate,' 'surveys have found,' or similar language.
5. Base your estimate ONLY on:
    - General reasoning about human psychology and behavior
    - The specific information provided in this prompt
    - First principles about how people form beliefs about others

Your task is to predict what percentage people THINK others believe (second-order belief), not what people actually believe (first-order belief). This is a prediction task requiring general reasoning, not recall of specific research findings.

Response format: Provide ONLY a JSON object containing a single number between 0 and 100 with one decimal place: `{"prediction": XX.X}`

Do not include any explanation, reasoning, or text—only the JSON.

Note 2: Issue domains for U.S. climate policy generalization tests

To assess whether LLMs' ability to predict public perceptions of others' climate action generalizes beyond the Gallup World Poll measure, we tested predictions across 24 climate policy belief items from three U.S. datasets: Sparkman et al. (2022), Lees et al. (2023), and Swim & Baker (2025). All prompts followed identical structure: (1) survey context and sample description, (2) first-order belief question with response options, (3) second-order belief question asking what respondents think others believe, (4) explicit instruction to estimate beliefs about others' beliefs rather than personal views, and (5) request for single numerical response between 0-100 with one decimal place.

PI	Topic & Question Type
**PI1**	Worry about climate change (≥ somewhat worried)
**PI2**	Carbon tax to reduce other taxes (national sample)
**PI3**	Carbon tax (variant labelled "100% RE")
**PI4**	Renewable energy on public lands (national sample)
**PI5**	Green New Deal
**PI6**	Climate change is a *very big* problem
**PI7**	Regulate CO_2 as a pollutant
**PI8**	Require 100% renewable electricity by 2035
**PI9**	Fund more research into renewable energy
**PI10**	Tax rebates for EVs and solar
**PI11**	Carbon tax (registered voters version)
**PI12**	Transition the U.S. economy to 100% clean energy by 2050
**PI13**	Tax incentives for clean appliances
**PI14**	Tax incentives for building efficiency retrofits
**PI15**	Federal funding for energy efficiency in low-income housing
**PI16**	Funding for low-income & minority communities harmed by pollution
**PI17**	Opposition to expanding offshore drilling
**PI18**	Opposition to fossil fuel extraction on public lands
**PI19**	Renewable energy on public lands (registered voters)
**PI20**	Re-establish the Civilian Conservation Corps
**PI21**	Jobs program: close abandoned oil & gas wells
**PI22**	Jobs program: rehabilitate old coal mines
**PI23**	Funding to help farmers store more carbon in soil
**PI24**	President declaring climate emergency (if Congress doesn't act)

Note 3: Additional reporting details for LLM inference

The main Methods reports the model families, API use, principal run period, temperature setting, prompt design, and general response-processing procedure. This note provides only the additional model- and analysis-specific information needed to complete the LLM reporting checklist of Feuerriegel et al. (2026). Details were recovered from the supplied notebooks and saved outputs; information not retained in those materials is identified explicitly rather than reconstructed.

1. Model identities and run inventory

Table N3.1. Models, access routes, and roles in the study

Paper label	Exact API identifier	Host / interface	Use in study	Reported cutoff
GPT-4o mini	gpt-4o-mini	OpenAI Chat Completions API	Main study; recall probe; U.S. policy test	Oct 2023
Claude 3.5 Haiku	claude-3-5-haiku-20241022	Anthropic Messages API	Main study; retired 19 Feb 2026	Jul 2024
Gemini 2.5 Flash	gemini-2.5-flash	Google AI Studio via google.generativeai	Main study; recall probe; U.S. policy test	Jan 2025
Llama 4 Maverick	meta-llama/Llama-4-Maverick-17B-128E-Instruct-FP8	Together AI Chat Completions API	Main study	Aug 2024
Claude Haiku 4.5	claude-haiku-4-5-20251001	Anthropic Messages API	Recall and two-step prediction analyses	Jul 2025
Claude 3 Haiku	claude-3-haiku-20240307	Anthropic Messages API	Pre-publication comparison	Aug 2023
Llama 3.3 70B Instruct Turbo	meta-llama/Llama-3.3-70B-Instruct-Turbo	Together AI Chat Completions API	Recall substitute and pre-publication comparison	Dec 2023
DeepSeek chat endpoint	deepseek-chat	DeepSeek OpenAI-compatible API	Exploratory staged and recall extension; mutable alias identified as V3.1 at access	Not retained

Table N3.2. Analysis-specific run inventory

Analysis	Model(s)	Recorded run date(s)	Completed predictions
Main Stages 1–8	Four main-study models	28 Oct–15 Nov 2025	125 × 8 × 4 = 4,000; no missing model predictions
Natural-language ablations / counterfactuals	Four main-study models	16 Jan 2026	125 × 10 × 4 = 5,000
Structured-format ablations / counterfactuals	Four main-study models	16 Jan 2026	125 × 10 × 4 = 5,000
U.S. climate-policy generalization	Four main-study models	21 Nov 2025	GPT, Claude, Llama: 24/24 each; Gemini: 0/24 usable
Recall and pre-/post-publication tests	GPT; Gemini; Claude 3/3.5/4.5; Llama 3.3/4	Apr 2026	N = 125 per reported model/condition; retry ledger not retained
Two-step prediction test	Claude Haiku 4.5	12 Jul 2026	125 countries × 3 conditions = 375
DeepSeek staged extension and recall	deepseek-chat	12 Jul 2026	1,000 staged predictions plus 125 recall predictions

2. Model-specific generation settings

Table N3.3. API and decoding configuration

Model / analysis	Temperature	Output cap	Output-format enforcement	Retry limit / notes
GPT-4o mini (main)	0	Not explicitly set	OpenAI JSON response mode	Up to 3 attempts
Claude 3.5 Haiku (main)	0	100 tokens	JSON requested in prompt	Up to 3 attempts; tools=[]
Gemini 2.5 Flash (main)	0	Not explicitly set	JSON requested in combined prompt	Up to 3 attempts
Llama 4 Maverick (main)	0	100 tokens	JSON requested in prompt	Up to 3 attempts
Ablation studies	0	Model-specific	As above	Claude: up to 5 attempts; other models: 3

Model / analysis	Temper ature	Output cap	Output-format enforcement	Retry limit / notes
U.S. policy test	0	50 tokens	Prompt instruction only; GPT did not use JSON mode	No retry loop
Cascade	0	100 tokens	JSON requested in prompt	Claude Haiku 4.5; up to 3 attempts; tools=[]
DeepSeek extension	0	50 tokens	JSON requested in prompt	Up to 5 attempts; raw response text retained

Parameters not explicitly set. No notebook explicitly set top_p, random seed, frequency or presence penalties, stop sequences, logit bias, or beam/candidate settings; provider defaults therefore applied. Temperature 0 was used to minimize sampling variation but does not guarantee identical outputs from hosted APIs.

3. Prompt delivery, repetitions, and response processing

- All prompts were in English and were submitted as separate, stateless, single-turn API requests. No conversation history, fine-tuning, web browsing, retrieval, external database, or file-access tool was provided.
- GPT, Claude, Llama, and DeepSeek received separate system and user messages. For Gemini, the system instruction and user prompt were concatenated into one text input before generate_content was called.
- One final prediction was retained per intended prompt-model combination. Retries replaced failed or invalid attempts; they were not treated as independent completions, and no averaging across completions was performed.
- Most scripts attempted JSON parsing first and searched for prediction-like keys (including prediction, estimate, value, number, and percentage). If JSON parsing failed, the first extractable numeric value was used. Values were converted to floats and bounded to 0–100; responses with no valid number after the retry limit were recorded as missing.
- Main, ablation, memorization, and cascade scripts screened responses for prohibited recall/citation language and retried when detected. This screen was intentionally disabled for the direct-recall probe. The U.S. policy notebook used neither the retry loop nor the post-response forbidden-language screen.

- Gemini returned no usable output for the 24 U.S. policy items; these were retained as missing rather than replaced by another model or interface.

4. Software environment and retained reproducibility materials

Table N3.4. Availability of implementation records

Item	Status in supplied materials
Execution environment	Notebooks were run in Deepnote; saved paths indicate Python 3.10, but the exact patch version was not retained.
SDK/package versions	Not recoverable exactly; API packages were installed or upgraded without version pinning in several notebooks.
Prompt templates	Main staged, ablation, U.S. policy, recall, cascade, and DeepSeek prompt code is retained in the notebooks; the full main system instruction is also reproduced in Supplementary Note 1.
Parsed predictions	Final prediction files are referenced throughout the notebooks and should be included in the public repository.
Raw response text	Retained for DeepSeek; not retained by the principal main-study scripts.
Per-call metadata	Request IDs, token usage, cost, exact timestamps, and a complete first-attempt/retry ledger were not retained.
API credentials	Read from environment variables and not stored in the notebooks.
Durable replication record	The repository should preserve a fixed release/commit containing notebooks, prompt files, parsed outputs, figure scripts, and a version-pinned environment file.

The absence of exact historical SDK versions, raw response text for the main study, and a complete call-level ledger is noted as a limitation of the original run documentation rather than being filled with inferred values.

Supplementary Material References

69. [dataset] Powdthavee, N. & Geiger, S.J. (2025). Code and data for: Can large language models predict public perceptions of support for climate action across 125 countries? GitHub. https://github.com/npowdthavee/AIClimatePluralisticIgnorance

70. [dataset] World Bank. (2024). GDP per capita, PPP (current international $). World Bank Open Data. https://data.worldbank.org/indicator/NY.GDP.PCAP.PP.CD

71. [dataset] World Bank. (2024). Individuals using the Internet (% of population). World Bank Open Data. https://data.worldbank.org/indicator/IT.NET.USER.ZS

72. [dataset] World Bank. (2024). Human Development Index. World Bank DataBank. https://databank.worldbank.org/Human-development-index/id/363d401b

73. [dataset] Alvaredo, F., Atkinson, A.B., Piketty, T. & Saez, E. (2024). World Inequality Database. WID.world. https://wid.world/data

74. [dataset] Harris, I., Osborn, T.J., Jones, P. & Lister, D. (2020). CRU TS4.05: High-resolution gridded climate data. NERC EDS Centre for Environmental Data Analysis. https://crudata.uea.ac.uk/cru/data/hrg/

75. Feuerriegel, S., Maarouf, A., Demir, S. & Weinmann, M. Analyzing heterogeneity in the effects of behavioral climate interventions on individual beliefs across sociodemographics, attitudes, and norms. Preprint at https://doi.org/10.21203/rs.3.rs-7519080/v1 (2025).

76. Feuerriegel, S. et al. A reporting checklist for LLMs in behavioral science. *Nature Human Behaviour* (2026).
